# A Smartphone-Based Crowd-Sourced Database for Environmental Noise Assessment

**DOI:** 10.3390/ijerph18157777

**Published:** 2021-07-22

**Authors:** Judicaël Picaut, Ayoub Boumchich, Erwan Bocher, Nicolas Fortin, Gwendall Petit, Pierre Aumond

**Affiliations:** 1 Centre for Studies on Risks, The Environment, Mobility and Urban Planning (CEREMA), Research Unit in Environmental Acoustics (UMRAE), French Institute of Science and Technology for Transport, Development and Networks (IFSTTAR), University Gustave Eiffel, F-44344 Bouguenais, France; ayoub.boumchich@ifsttar.fr (A.B.); nicolas.fortin@univ-eiffel.fr (N.F.); pierre.aumond@univ-eiffel.fr (P.A.); 2 Lab-STICC CNRS UMR 6285, IUT de Vannes, 8 Rue Montaigne, BP 561, CEDEX, F-56017 Vannes, France; erwan.bocher@univ-ubs.fr (E.B.); gwendall.petit@univ-ubs.fr (G.P.)

**Keywords:** environmental noise, crowd-sourcing, smartphone application, data analysis

## Abstract

Noise is a major source of pollution with a strong impact on health. Noise assessment is therefore a very important issue to reduce its impact on humans. To overcome the limitations of the classical method of noise assessment (such as simulation tools or noise observatories), alternative approaches have been developed, among which is collaborative noise measurement via a smartphone. Following this approach, the NoiseCapture application was proposed, in an open science framework, providing free access to a considerable amount of information and offering interesting perspectives of spatial and temporal noise analysis for the scientific community. After more than 3 years of operation, the amount of collected data is considerable. Its exploitation for a sound environment analysis, however, requires one to consider the intrinsic limits of each collected information, defined, for example, by the very nature of the data, the measurement protocol, the technical performance of the smartphone, the absence of calibration, the presence of anomalies in the collected data, etc. The purpose of this article is thus to provide enough information, in terms of quality, consistency, and completeness of the data, so that everyone can exploit the database, in full control.

## 1. Introduction

Noise is a very significant source of pollution, particularly in urban areas, with significant effects on health. The fight against noise is a fundamental societal and health issue, to which the public authorities are trying to respond by putting regulations in place. In Europe, for example, the directive 2002/49/EC aims to establish an inventory of noise nuisance, to propose actions to reduce nuisance and to communicate to citizens about their exposure to noise [1]. In this regulatory context, the main tool for decision-makers is the production of strategic noise maps.

These maps are generally produced using specific software, integrating noise emission and acoustic propagation models, coupled with geospatial data and traffic information. Although these maps are limited by the calculation assumptions and the quality of the input data, they make it possible to assess the broad outlines of a noise distribution in a city and to evaluate the effect of action plans to reduce noise. However, they generally lack realism, particularly from the point of view of the temporal dynamics of noise. Conversely, noise observatories, consisting of a large number of acoustic sensors, offer a more realistic description of noise environments. However, the limitation of the number of sensors, for technical and cost reasons, does not allow carrying out noise mapping with a sufficient spatial step.

Faced with these observations, alternatives have been proposed. In particular, the use of more affordable sensor networks has been investigated, allowing to densify the observation points [2]. Another way consists in the involvement of citizens as data collectors, in a crowd-sourcing approach. For example, the Smart Citizen System project proposes a low-cost sensor specifically dedicated to collect noise data by citizen action [3]. Considering a soundscape approach, data produced by people on location-based social networks can also be analyzed to produce maps of the sound environment, like with the Chatty maps experiment [4] or more recently by Gasco et al. [5]. The Sound Around You project is another example, by proposing a web interface to collect soundscape recording and opinions [6]. Nowadays, among all the citizen science-oriented approaches, the one based on the use of smartphones is undoubtedly the most developed in the literature. In particular, Santini et al. have demonstrated the capabilities of a smartphone to perform environmental acoustic measurements [7]. It was followed by several works that have given rise to specific noise and soundscape crowd-sourcing type applications and platforms (see Ear-Phone, NoiseSPY, and NoiseTube applications, respectively, in [8,9,10]). What is interesting in these first works is that, despite the technical limitations of the time (i.e., smartphones with limited technical capabilities and resources), almost all the topics related to this issue had already been discussed: smartphone calibration, data quality, noise maps reconstruction, contextualized data collection (perceptual data), the need for a complementary web interface, the need to know the context of the measurement, the implementation of specific events to organize data collection, contributors privacy, motivation of contributors, etc. Subsequently, other contributions have appeared on this subject; the reader may refer to recent literature reviews [11,12,13,14,15] for more details.

The evolution of Information and Communication Technologies (ICTs) and the experience obtained from the past researches allowed the implementation of very advanced solutions, in the last few years, among them the Sense2Health platform (which led to the Ambiciti plateform) [16], integrating a data assimilation model to produce more realistic noise maps; the Hush City platform [13], for collecting data in quiet areas; the City Soundscape platform [17], with the objective to evaluate action plans for road noise reduction; and the GRCSensing platform [14] with an interesting feature for distributing tasks to users in order to capture noise in specific urban areas and times.

Proposed more recently, the NoiseCapture project is completely in line with the last platforms [18], but extends the concept of participatory science to that of open science. Thus, all source codes, whether for the smartphone application, the spatial data infrastructure, or the web interface, are released as open source. In the same way, the data are available in open data in many ways, and, as far as possible, the scientific productions, in open access. Attention was also paid to the long-term sustainability of the system, the NoiseCapture project being part of an operational framework and not in the form of a short-term experimentation. The objective is to ensure a collection of data over several years, in order to constitute a reference database for the study of sound environments over the long term. The respect for privacy and use of personal data is also a founding element of the NoiseCapture project; in order to respect the national regulation, in particular in Europe, no sound or video recordings are made, nor is any personal information collected; the use of the application does not require the creation of an account. Finally, the developers of the application have paid great attention to the quality of the acoustic data collected, by integrating proven signal processing algorithms, and by proposing several methods for smartphone calibration. After more than 3 years of existence, the amount of data collected worldwide thanks to the application is thus considerable (more than 100,000 downloads, 74,000 contributors, 260,000 tracks that represents around 60 million of one second measurement points), showing the interest of the citizens for this participatory approach and offering very promising operational and research perspectives.

Nevertheless, the exploitation of the database, whether in an operational or research context, requires a good knowledge of the inherent limitations of the methodology, such as the lack of control of the measurement protocol, poor acoustic calibration of the application, measurements tainted by uncertainties, the misuse of the application, the metrological limitations of smartphones, the context of the measurement, etc. In order to ensure that any user of the database has a perfect knowledge and control of the information contained in the database, a full description and an analysis of the database is performed in this article, in order to highlight the various uncertainties, irregularities, or inconsistencies that need to be considered before any exploitation of the data. This analysis also highlights future evolution that it would be interesting to consider in order to improve the application and to increase the quality of the collected data but also to collect additional information in order to better take into account the context of the measure in its exploitation. This article does not therefore constitute an acoustic study of sound environments, but provides a framework for understanding the NoiseCapture database for its future exploitation. The study of the noise environments using this database will be the subject of further works.

The NoiseCapture platform is first presented in Section 2. The collected data are then described and analyzed in the Section 3, providing sufficient information to a future user, for an exploitation of the database in total control of the nature of the data and their possible limitations. In Section 4, a discussion is provided for improving the application and the methodology to increase the data quality and analysis. Last, Section 5 concludes this work.

## 2. NoiseCapture Application and Database Description

### 2.1. NoiseCapture History

The development of the NoiseCapture application was initiated by the french National Center for Scientific Research (CNRS) and the Université Gustave Eiffel (formerly Ifsttar) within the framework of the European ENERGIC-OD project [19], which aimed at producing and redistributing geospatial information in open data to user communities. The development continued thereafter, as a part of the Noise-Planet project [20], with the objective to combine geomatic and acoustic sciences for the evaluation of outdoor sound environments. In line with the general goal of the Noise-Planet project, it was decided to develop the NoiseCapture application in the framework of an Open Science approach, with the dissemination of source codes in Open Source, data in Open Data, and as far as possible, scientific dissemination through publications in Open Access journals.

The initial objective of the NoiseCapture application was to propose a smartphone application to a community of specialists (technical staff within a local authority for example), in order to assess the outdoor sound environments in their territory, by using a collaborative mapping tool. The target audience was therefore initially people with technical and, possibly, acoustic knowledge, allowing them to understand a rather professional smartphone application.

The NoiseCapture application was designed in order to carry out acoustic measurements over a shorter period of time, if possible while walking, in order to collect data on a large spatial area. The user was expected to keep the smartphone in hand throughout the measurement, especially to control the measurement. Thus, the measurements are user-initiated and not background. Each user can then decide to upload data to a remote server that collects all the data in a database, performs further analysis, and represents the results collected by a set of users in the form of a noise map.

The choice of the development environment was oriented towards the most widespread platform, namely, Android, whose market share has been above 80% for many years (around 15% for iOS (a mobile operating system created and developed by Apple Inc.)) [21]. The porting of the application to iOS has not been achieved, although a gain in terms of metrological quality may be possible due to a lower variability in devices [22]. In order to promote the diffusion of the application worldwide, the application has been translated thanks to volunteers, in several languages (English (en), Chinese (China, zh_CN), French (fr), Greek (el), Polish (pl), Portuguese (Brazil, pt_BR), Spanish (es)). In the rest of this article, the terms and features of the application refer to the 1.2.15 version (release 51) of NoiseCapture with the default language (i.e., “en” for English). The last public NoiseCapture release is available on Google Play (“Google Play” brand is property of Google LLC) [23].

### 2.2. NoiseCapture Description

#### 2.2.1. NoiseCapture Android App

**Figure 1:**
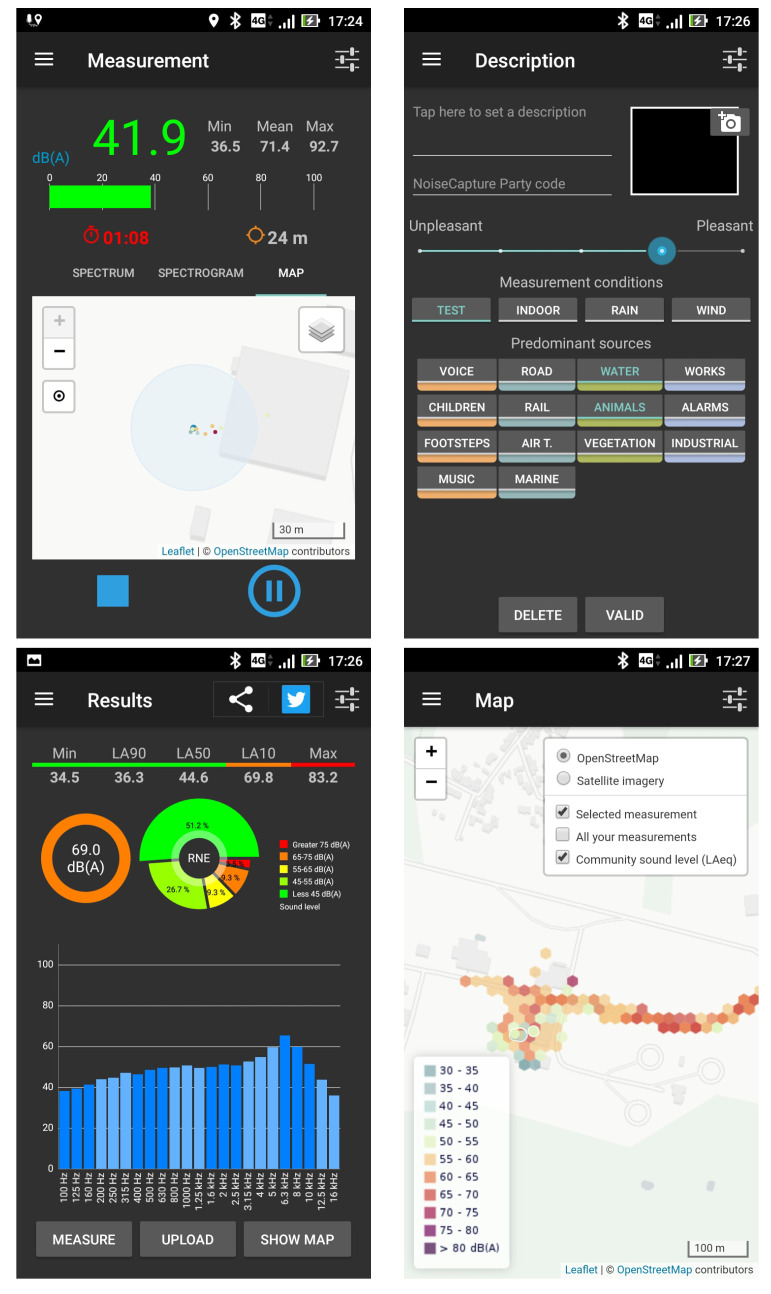
NoiseCapture Android application main screens. From **top**/**down** and **left**/**right**: Measurement, Description, Results, Map.

From a functional point of view, the NoiseCapture application uses the principles of a “pocket” sound level meter. The main screen (Figure 1, “Measurement”) presents the results of an acoustic measurement through several classical acoustic indicators: an instantaneous sound level (calculated on a sliding window), as well as the minimum (Min), maximum (Max), and average (Mean) instantaneous sound levels over the duration of a measurement. The instantaneous spectrum by third octave band between 100 Hz and 16 kHz is also proposed on a specific tab, as well as a spectrogram. The duration of the measurement is also indicated: the user can start, pause/resume, and stop the measurement at their convenience, and the measurement duration can also be automatically be fixed in the application settings (the user starts the measurement, but it stops by itself after a certain duration).

In accordance with the initial objective of the application, i.e., the production of a noise map, each measurement is geolocalized with the last known GPS location. The measurement screen also indicates the position of each measurement, every second (i.e., the “Measurement Point”), and more globally the trace of a measurement (i.e., the “Measurement Track”) according to the user displacement. The accuracy of the location is also indicated both numerically and graphically on the map. At this point, note that the acoustic indicators that are calculated and displayed on this screen, as well as those that are presented on the “Results” screen, do not result in any audio recording; these indicators are calculated on the fly.

After a measurement has been performed, the user accesses a second screen (Figure 1, “Description”), which allows the user to give additional information to the measurement. Filling this form is entirely optional. Some information, such as “Description” and “Pictures”, are only stored on the smartphone, while other data may be collected and transmitted to the NoiseCapture remote data server. The choice of whether or not to transmit the measurements and information can be configured by the user. On this screen, 3 types of information can be provided: (1) information on the perceived quality of the sound environment (“Pleasantness”); (2) information on the measurement conditions using 4 tags (“Test”, “Indoor”, “Rain”, and “Wind”); (3) information on the nature of the sound sources perceived during the measurement, using 14 tags (“Footsteps”, “Voice”, “Natural”, “Mechanical”, “Human”, “Works”, “Air t.”). (i.e., “Air Traffic”), “Entertainment”, “Children”, “Music”, “Road”, “Rail”, “Marine”, “Alarms”, “Industrial”, “Water”, “Animals”, “Vegetation”).

Once this optional information has been validated, the user has access to a summary of the measurement in the “Results” screen (Figure 1). Acoustic indicators are specific to the evaluation of outdoor sound environments, based on 1 s average sound level [24], such as noise levels in percentiles ($L_{A10}$, $L_{A50}$ and $L_{A90}$), maximum (Max) and minimum (Min) values as well as the average sound level in dB(A) and the average spectrum over the measurement time. In addition, a graphical representation, noted RNE, shows the distribution of the 1-second noise levels.

A “Map” can also be displayed on a specific screen (Figure 1) in order to locate the measurement points and to represent the average values shared by the user community and aggregated by the NoiseCapture remote server. Other functionalities are also offered by the application, such as smartphone calibration and data archiving, but are not detailed in the present paper. More details are given in the following reference [18].

#### 2.2.2. NoiseCapture Web Interface

While the NoiseCapture application can be used to meet the need of a user (i.e., to assess a noise level in his own environment), the overall interest of the approach lies in the sharing of data within a community, which requires the data to be centralized on a remote server. To this end, a Spatial Data Infrastructure (SDI), called OnoMap, has been specifically implemented to propose 3 functionalities: to (1) collect, (2) display, and (3) share all the data produced by the contributors [20,25,26].

**Figure 2:**
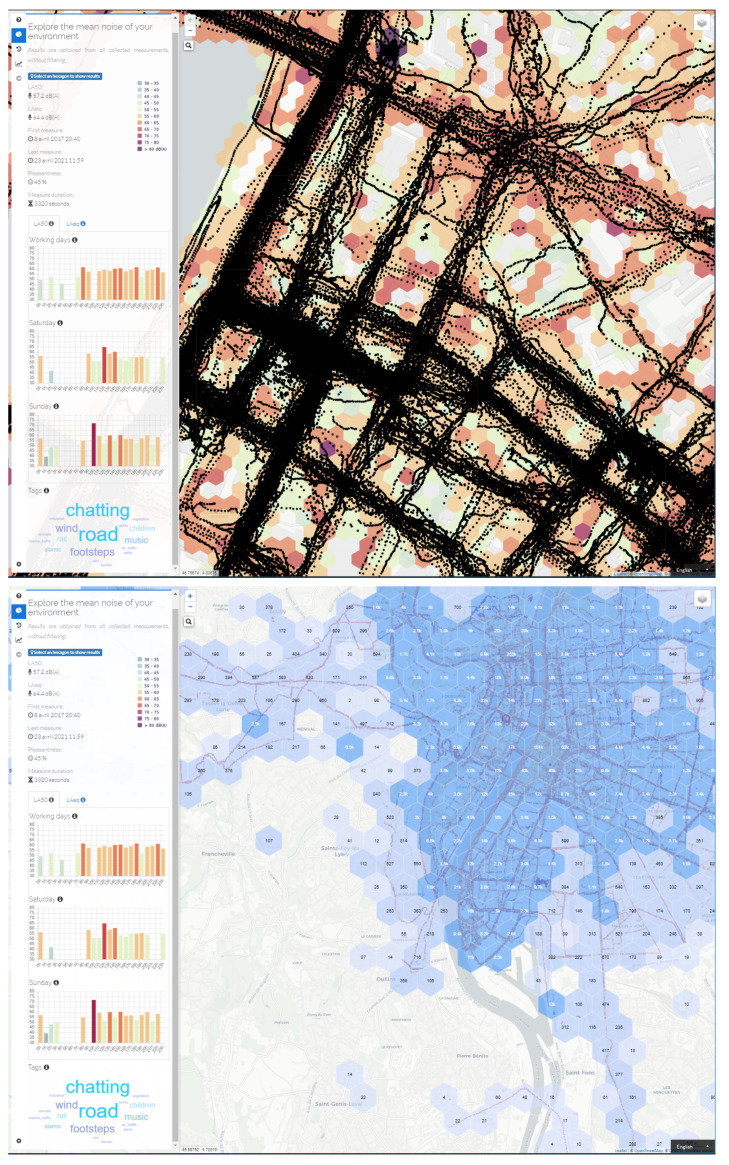
Screenshots of the NoiseCapture map website. Example of collected data representation, on the Lyon French city (from https://noise-planet.org/map_noisecapture/index.html#18/45.75387/4.84052/ (accessed on 15 July 2021)), at a higher (**up**) and lower (**down**) zoom levels.

The second functionality is the most visible part of this SDI, as it allows to display the collected data to any visitor of the website, in an aggregated and understandable form. Figure 2 illustrates an example of a graphical representation, centered on the city of Lyon in France. Depending on the zoom scale of the map, the main window presents the collected data either in a numerical form, in terms of number of points per geographical area (represented by hexagons of different sizes depending on the zoom level), or in the form of a ‘classical’ noise map. In the latter representation, only certain acoustic indicators are presented, by aggregating all the values collected over a fixed spatial extent (i.e., average of an acoustic indicator in a hexagon). The left-hand side of the web page gives access to additional contents, such as the history of the last 30 series of measurements (almost in real-time), general statistics on all the data collected (most contributing countries, number of measurements, most used tags, etc.). By clicking on a hexagon on the noise map (at the highest zoom levels), it is also possible to access to more detailed information, such as the number of points and the total duration of measurements in the corresponding hexagon, the average equivalent sound level ($L_{A,\mathrm{eq}}$ and LA50), the tags used (in the form of a tag cloud), as well as the hourly distribution of sound levels on different days of the week. All the information presented in this web page results from a direct exploitation of the NoiseCapture database, and illustrates some relatively simple analysis. Downloading the collected data (the third functionality of the SDI) offers many more perspectives of analysis and representation of the data. The upper screenshot of Figure 2, which displays the position of the measurement points, underlines again the interest of the method and the very rich perspectives of analysis of the sound environments, with regard to the quantity of data that can be collected on a given spatial extent. The purpose of this article is precisely to propose a first analysis of these raw data, in Section 3, so that they can be exploited, in a second step, to perform a relevant sound environment analysis.

### 2.3. NoiseCapture Raw Database

The analysis that is carried out thereafter covers the data collected since the official publication of the application on 29 August 2017 until 28 August 2020 (3 years). These collected data cover several releases of the application (from 28 to 51, see Table 1), some of which make changes to the nature of the data collected and the features. Previous release (before release 28) and intermediate pre-releases correspond to beta versions, published on Google Play to a specific panel of testers. The database available for download may include data from beta and pre-release versions; it may be useful to filter these data both on a period (from the launch of the application) and on the version (since release 28), for a relevant analysis.

### 2.4. NoiseCapture Installs and Uninstalls

As mentioned above, the initial audience targeted during the development of the application was primarily technical staff, with sufficient expertise to be able to use the application in satisfactory conditions (compliance with a measurement protocol, acoustic calibration of the smartphone, critical analysis of the measurements, etc.). The production of data was therefore initially part of a supervised activity with a professional purpose. In practice, the publication of the application on Google Play, combined with an institutional communication, was relayed by the national and then European media, generating the interest of a wider public than initially foreseen. Very quickly, the application was then downloaded in other countries, notably the United States, by a large audience. This confirms once again the interest of citizens and communities in the issue of noise environments and reaffirms the major societal challenge of research on this subject.

**Figure 3:**
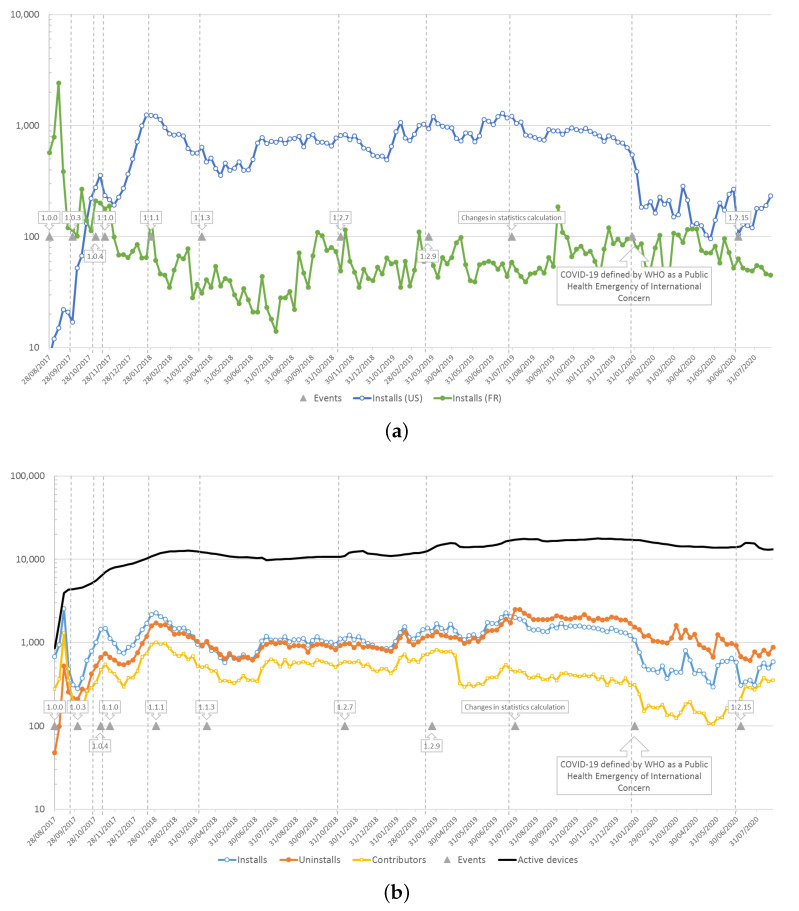
Weekly evolution of user installs and uninstalls of the NoiseCapture application (data from Google Play dashboard): (**a**) global data and (**b**) data for France and United states of America. ‘Installs’: number of users who have installed the application at least on one device; ‘Uninstalls’: users who have uninstalled the application from all their devices; ‘Active devices’: number of active devices that contains application, and which was turned on at least once in the previous 30 days; ‘Contributors’: Users who have upload data to the NoiseCapture remote server; ‘Events’: Events that may have a particular impact on the users behavior. (**a**) Application installs for France (FR) and United-States of America (US); (**b**) Application installs/uninstalls/contributors.

Figure 3a illustrates the number of installs of the application for the two countries (US and FR) that contribute the most to the data collection today; these data are obtained from the application dashboard on Google Play. This figure clearly shows the impact of the launch of the application in France, with a high number of installs in the first few weeks, followed by a decrease to an average level of about 60 installs per week; conversely, there is a gradual increase in the number of installs in the US, to an average level of 800 installs per week. From a global point of view, Figure 3b shows a certain stability around the 1000 weekly installs worldwide, over most of the period concerned. Unsurprisingly, the uninstalls rate follows the rate of installations, but the number of uninstalls tends to exceed the number of installations since the end of 2019, which leads to a decrease in the number of active devices (i.e., devices having installed the application and being turned on over a 30-day period), which has gone from about 17,000 at the end of 2019 to 13,000 at the end of 2020.

**Figure 4:**
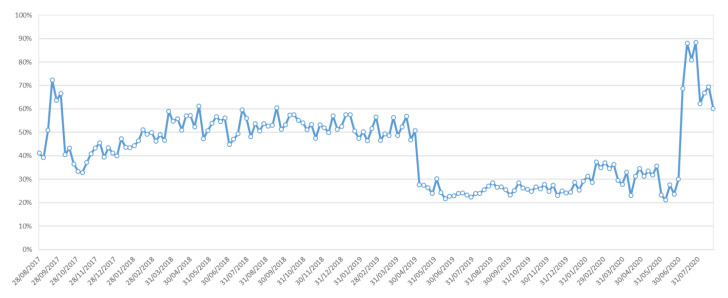
Weekly ratio between the number of contributors to the NoiseCapture database and NoiseCapture installs.

Even if it is difficult to make a direct link between the number of installations and the number of different contributors, we can see that on average, about 50% of new installations give rise to at least one contribution on the NoiseCapture server over the period from 2017 to mid 2019 (Figure 4). The ‘break’ that is visible on this figure in mid-2019, due to a very sharp drop in the number of contributors (Figure 3b), is at this stage undetermined. Conversely, Figure 3b shows the interaction between certain events quite well, in particular the impact of the publication of a new release on the number of new installs (also visible on the number of active devices). For example, the decrease in number of installs and contributors observed from the beginning of 2020 coincides with the beginning of the COVID-19 pandemic, particularly visible in the US community (Figure 3a); this is not visible with the French data, but we can quite imagine that there is a link between these two events. A detailed analysis of COVID-19 lockdown and user behavior in each country would undoubtedly lead to some hypotheses. This shows again the interest of such alternative way for collecting data for the study of the noise environment.

## 3. Analysis of the Collected Data

### 3.1. Collected Data

As mentioned above, the statistical analysis on NoiseCapture data presented in this section involves data collected from 29 August 2017 to 28 August 2020 (3 years of data). During this period, the NoiseCapture application has proven to be successful to perform and gather acoustics measurements. NoiseCapture has been downloaded more than 160,000 times on Google Play [23], with 76,229 contributors to the database all over the world. Approximately 91.7% of the users present in the database (69,898 of the 76,229 contributors in the present database) have contributed within this period. Table 2 shows that 260,422 tracks (59,685,328 points) have been collected, with an average of 229.2 points (i.e., seconds) per track (a median value of 28 points per track).

All the collected data during the corresponding period of analysis have been integrated into a spatial relational PostGIS database [29] (i.e., a spatial database extender for PostgreSQL object-relational database [30], adding a support for geographic objects). The database is fully available for download [31] and can be used according to the OdBL license [32]. It is important to specify that all the data integrated in this database fully respects the privacy of users as no personal data is collected.

**Figure 5:**
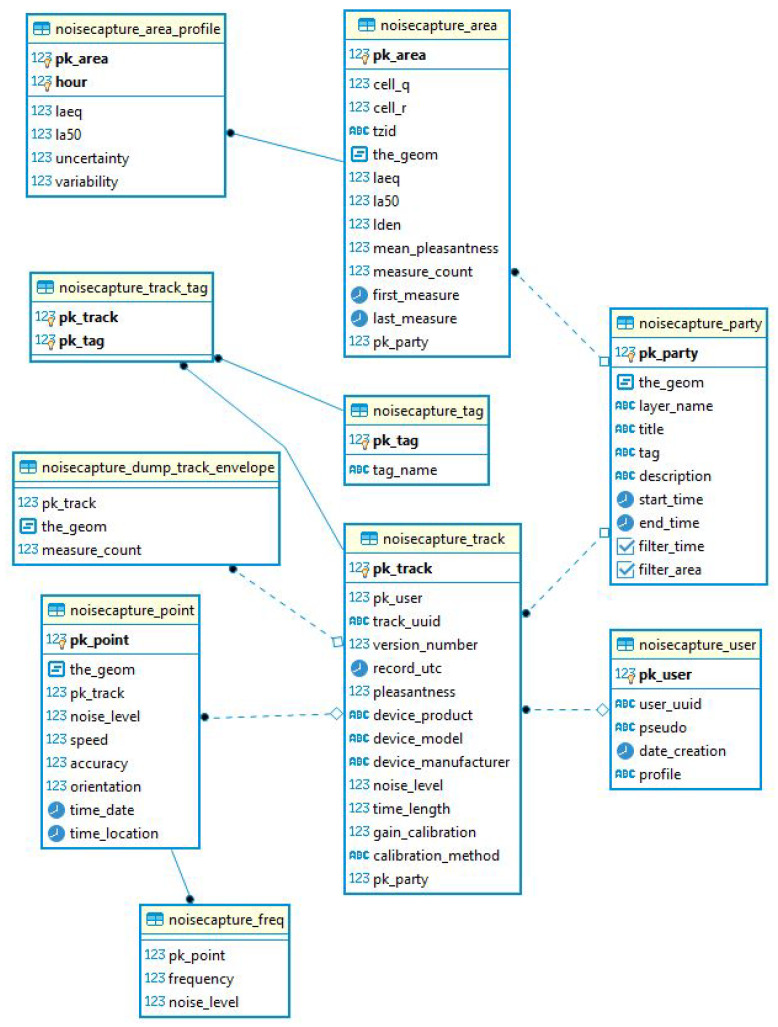
Entity relation diagrams (ERD) of the NoiseCapture PostGIS database. The type of each field in the tables is mentioned: ‘123’ for ‘float’ values, ‘ABC’ for ‘text’ chains, ‘timestamptz’ for time stamp date, ‘🗹’ for ‘boolean’ value. The ‘key’ yellow symbol is used to display primary keys of the table, whose corresponding names are displayed in bold.

The data collected from smartphones are organized into several tables (Figure 5):

- For each measurement ‘Point’ (i.e., a measurement performed every second during a ‘Track’), the global ‘noise_level’ value measured at the measurement date ‘time’ is given in the ‘noisecapture_point’ table. In addition, the ‘speed’ at the measurement point, the geolocalization (‘the_geom’), the date of the localization (‘time_location’), the ‘accuracy’ of the geolocalization as well as the smartphone ‘orientation’, all obtained by the smartphone GPS, are given. In this table, the measurement point is defined by a primary key ‘pk_point’ (generated by the database) allowing to make the relation with two other tables ‘noisecapture_freq’ and ‘noise_capture_track’ (via the primary key ‘pk_track’);
- The ‘noisecapture_freq’ table contains for the measurement point defined by the primary key ‘pk_point’, the ‘noise_level’ spectrum by third octave band ‘frequency’ between 100 Hz and 16 kHz;
- The ‘noisecapture_track’ table contains all the information associated with a measurement corresponding to a set of measurement points. Each measure is defined by a primary key ‘pk_track’ (generated by the database) and a unique identifier ‘track_uuid’ (generated by the application). Each measurement contains the following information: the user primary key ‘pk_user’, the release number of the application ‘version_number’, the characteristics of the smartphone (the reference ‘device_product’, the model ‘device_model’ and the manufacturer ‘device_ manufacturer’), the date of the start of the measurement ‘record_utc’, the duration ‘time_length’ of the measurement, the average sound level over the duration of the measurement ‘noise_level’ and the perception of the sound environment ‘pleasantness’. Information on the acoustic calibration of the smartphone is also associated with the measurement: the choice of the calibration method ‘calibration method’ and the corresponding calibration value ‘gain calibration’. Finally, if the measurement was performed during a NoiseCapture Party (see Section 3.6), the corresponding code is indicated in the value ‘pk_party’.
- The ‘noisecapture_user’ table gives for each primary key ‘pk_user’, the user identifier ‘user_uuid’ (this unique identifier is randomly created each time the application is installed on a smartphone), the user creation date ‘date_creation’ (created by the remote server when uploading the data, not at the application installation), as well as the user ‘profile’ defined by the choice of a value in a list, as ‘EXPERT’, ‘NOVICE’, and ‘NONE’. The value ‘pseudo’ in the table has been created for future functionalities and is currently not used.
- The ‘noisecapture_track_tag’ table contains for each measure defined by the primary key ‘pk_track’, the list of tags selected by the user to describe the sound environment. The identifiers of the corresponding tags are defined in the value ‘pk_tag’. The correspondence between the identifier of the ‘pk_tag’ tag and the name of the tag (‘tag_name’) is defined in the ‘noisecapture_tag’ table.
- The ‘noisecapture_party’ table contains information about the realization of the NoiseCapture Party events [33] (see Section 3.6 for details). In principle, such event is supervised by an expert, over a limited duration and spatial extent, allowing to generate a series of measurements. It can for example be an action carried out by a Community in order to carry out a series of measures concentrated in a particular district. A NoiseCapture Party has much the same objectives as an OpenStreetMap (OSM) Mapping Party to feed the OSM global database [34]. This table gives for each NoiseCapture Party, a specific primary key ‘pk_party’ (generated by the database) returning the code of the NoiseCapture Party (‘tag’), the title ‘title’ and a description ‘description’, the spatial extent defined by a geometry ‘the_geom’, the start and end dates of the event ‘start_time’ and ‘end_time’. The boolean values ‘filter_time’ and ‘filter_area’ are used to define whether the collected data are integrated into the NoiseCapture Party set, whether or not the measurements have been made with the right NoiseCapture Party code, but outside of the temporal and and spatial limits. The value ‘layer_name’ is only used to give a name to the corresponding map layer in the web page displaying the data on the corresponding website [26]. It is important to specify that the NoiseCapture Party is technically created by the people in charge of the development of NoiseCapture. If an invalid value is used for the NoiseCapture Party code field in the ‘Description’ screen of the application (Figure 1), the code is removed, but the corresponding data are still included in the database.

### 3.2. User Information

#### 3.2.1. User Profile

The use of the application according to the respect of technical procedures in acoustics is an important issue for the quality of the produced data. In order to have information on the user experience, at the installation step of the application, the user is asked to define his expertise using a 3 levels scale: ‘EXPERT’, ‘NOVICE’, or ‘NONE’. Analyzing the 76,229 different contributors in the database, over the period from 29 August 2017 to 28 August 2020 (using the field ‘date_creation’, which corresponds to the date of creation of the user in the NoiseCapture database on the remote server), 10.19% defined themselves as ‘EXPERT’, 24.78% as ‘NOVICE’, and 64.17% as ‘NONE’. A very large majority of contributors therefore have no experience in the field, which can necessarily lead to a bias in the quality of the data collected. This is an expected behavior for a citizen science project.

Note that for 653 contributors (0.86% of the total number of contributors), the profile field is empty, meaning that the information is not available during this period. This is due to an update of the application from a version prior to version 28 (the field ‘user_profile’ has been integrated from version 28), as the user is not asked to modify this field during an update. In detail, the analysis of these cases shows that most of the concerned contributors (641) were declared in the database in the first 2 months after the launch of the application, while the other 12 contributors were declared during the rest of the period.

#### 3.2.2. User Devices

For this type of measurement application, the metrological quality of the device, whether for acoustic measurement or for other data (GPS and other sensors), is an essential aspect. On this point, the identification of the smartphone can provide useful information for a later analysis of the collected data, in postprocessing. Among the possible treatments, an *a posteriori* calibration of the acoustic data, for example, based on a smartphone knowledge base, offers interesting prospects for improving the quality of the acoustic indicators produced by the application [35,36]. Some works have also shown that the knowledge of the manufacturer can provide a useful information on the accuracy of the measurement [37]. This justifies the need to collect hardware-related information, namely, the ‘device_product’, the ‘device_model’, and the ‘device_manufacturer’, defined by Android documentation as the name of the overall product, the end-user-visible name for the end product and the manufacturer of the product/hardware respectively [38].

As an example, considering the Samsung Galaxy A10, which is one of the best selling Android phone, the device field will give the data of Table 3. This table shows that the commercial name of the corresponding smartphone can be declined in several device models that most of time refer to distinct version (’A10E’ for ‘SM-A102’, ‘A10’ for ‘A105’, ‘A10S’, for ‘A107’) or to the international region where they were deployed.

Over the period in question, the database references 646 distinct manufacturer names. However, the same manufacturer can appear under a different spelling; this is the case, for example, for Samsung, appearing with the following names: ‘samsung’, ‘Samsung’, and ‘SAMSUNG’. By grouping the manufacturers without taking into account the sensitivity to upper and lower case, one can identified finally 520 manufacturers (Table 4) with 5300 different smartphone models. Nevertheless, three manufacturers alone (Samsung, LGE and HUAWEI) account for about 35.2% of the models, and cumulate nearly two thirds of the tracks (65.1% or 66.3% in number of points). The top 15 manufacturers account for 90.3% of the tracks (91.1% of the points).

**Figure 6:**
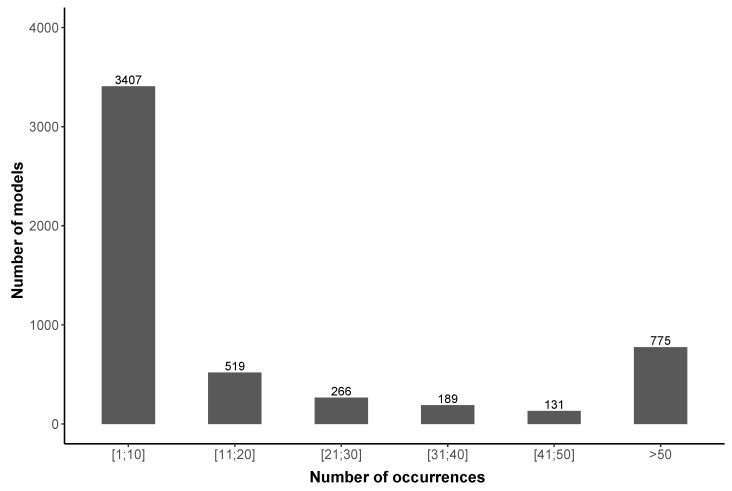
Distribution of the number of occurrences of a smartphone model in the database. As an example, 775 device models appear more than 50 times in the database.

The distribution of measurements is more important in number of models (Table 5), as the top 15 models only have 15.9% of tracks (16.7% of points), each model accounts to only between 1.8% and 0.8% of the whole measurements. To reach half of the tracks, we have to consider 130 different models, and 1077 models to exceed 90%. In addition, Figure 6 shows that most of devices appears only few times in the database; for example, 3407 different devices are used 10 times or less; conversely, only 775 device models appear more than 50 times in the database. In detail, there are 1228 smartphones that are used only once and 677 twice.

By focusing on the two most contributing countries (US and FR, Table 6), we find a consistency between the manufacturers market share and the brands most represented in the database. This table also shows that Apple with the iPhone model (Apple and iPhone are trademarks of Apple Inc.) is a very important manufacturer in the US and in France; it suggests that the current NoiseCapture database excludes a very large number of users, that in the case of iOS users, represents a specific segment of the population, considered with higher income and education levels, in-app engagement [39]. The initial choice to select Android as the only development platform, as it represents a global market share of 80%, can thus be questioned. It would seem wise to consider an additional iOS version of the application in the future, considering the user audience, but also metrological considerations.

#### 3.2.3. User Contribution

Table 7 illustrates the use of the application in terms of number of contributions. Slightly more than half of the contributors have contributed to the database only by 1 track, and nearly 95% by less than 10 tracks. It is likely that most of the contributors concerned by only few contributions were just interested by testing the application, before either uninstalling it or putting it aside. This table also shows that there is a small proportion of contributors who have collected a very large number of tracks, up to several thousand for some. It seems obvious that these contributors have integrated themselves into an active approach to collect measurements and that this type of user is the most interesting part of the community, *a priori* motivated by the collaborative approach. The animation of this specific community must be a priority in the future. This last point will be discussed in Section 4.

**Figure 7:**
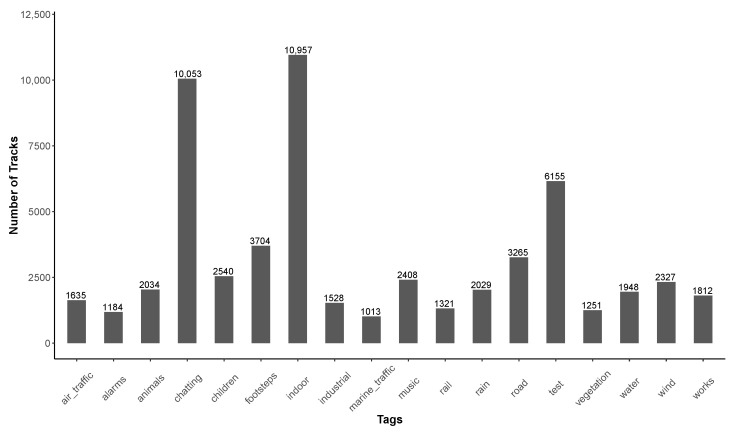
Distribution of the tags used for tracks collected by user who have 1 only contribution to the database.

Considering the contributors with only one contribution, Figure 7 shows that 6155 of them (16.9%) have used the “test” tag, meaning that they were just testing the application. In addition, Table 8 shows that 14,034 (38.5%) of these “one-shot” use of the application have duration less than 20 s. These two observations may partially support our hypothesis that these one-shot contributors just want to test the application, and probably do not plan to use the application again.

Table 9 shows that for users that realize more than one contribution, the second contribution comes in the next 4.4 days, on average. However, for the major part of the contributors (158,631, 83.3%), the second contribution is realized in the same day, and in the same week for 9.9% (18,971).

### 3.3. Measurement Geolocalization

#### 3.3.1. Geolocalization

In this paragraph, we present statistics and information related to the geolocalization of the NoiseCapture data. The variable ‘the_geom’, which gives the coordinates of the measurement point in the WGS 84 (EPSG:4326) map projection, has been used to perform this study.

As the country of the measurement is not in the data set, the following 2-step process has been carried out in order to define the country of origin of each measurement. First, a table called ‘noisecapture_track_frame’ was created using the PostGIS/PostgreSQL function ‘ST_EXTENT()’ [41] that returns a box that bound each track. Second, a table called ‘gadm’, mapping the administrative areas of all countries [42], has been used to create a table called ‘noisecapture_country_track’ by associating each track to the first country that contains the track bounding box.

Table 10 shows that the United States contributes more than third of NoiseCapture database, while France contributes approximately 10% of track data (8.3% point data). A strong French contribution was obviously expected, the application having been developed by French research institutes, and also because of a strong relay by national media. Conversely, it is difficult to explain the large amount of data produced by the US, except to consider that this country has a high population (3rd in the world in 2020, [43]), compared to France (22nd in the world). In addition, because some countries do not have access to Google Play or use alternative app stores, and since the NoiseCapture application is only available on Google Play store, it is not surprising that they are not found as a data producer. This is the case of China (Google Play not available in China) and Russia (an alternative app store is mainly used), for example, while they represent an important part of the world’s population (1st and 9th in the world, respectively).

Although the ranking of Peru and the Netherlands in terms of population is low, these two countries are in the top 5 in terms of number of tracks (Table 10). For these countries, the number of tracks compared to the number of contributors is very high (especially for Peru), which highlights an intensive measurement activity, which is perhaps part of a voluntary and organized action (like a NoiseCapture Party for example). A spatio-temporal analysis of the data produced in these two countries, as well as a detailed analysis of the behavior of the corresponding contributors, could eventually provide some answers. More globally, the implementation of cluster detection techniques could be an interesting way to identify organized events.

While conducting the study, it was observed that 32.5% of the tracks contain points without geolocalization (i.e., the field the_geom is empty), which represents a total of 10,783,609 points (18% of the total number of points), distributed over 141 countries. For 75% of these tracks (63,538 tracks), all the corresponding points are concerned by a lack of geolocalization (i.e., the whole track can not be geolocalized). The main reason is that the geolocalization has not enabled on the smartphone. By further analyzing, it was also observed that a large part of these tracks correspond to indoor measurements (20,045 (7.7%) of the corresponding tracks are defined with the ‘indoor’ tag). Tracks with a partial lack of geolocalized points may be due to a local loss of GPS localization, for example, when passing through a tunnel. A spatial analysis crossed with other geographical data can possibly bring elements of answer in this case.

Even when the measurement points are localized (i.e., the GPS actually transmits a position), this measurement can have a poor accuracy. Putting aside the technical quality of the hardware used in the smartphone for GPS location, this poor accuracy may be obtained when the measurement is made in an environment that is not clear enough (in or near a building, overcast sky) making it difficult to connect to a sufficient number of GPS satellites.

#### 3.3.2. Accuracy

**Figure 8:**
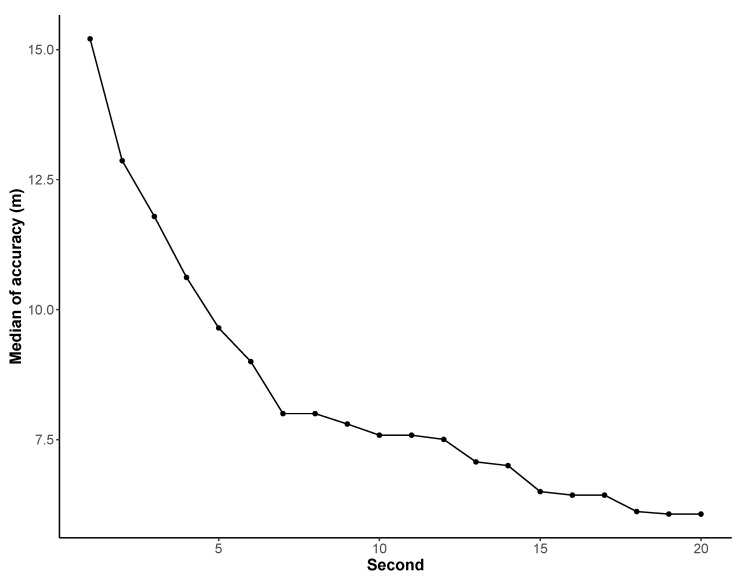
Evolution of the median value of the accuracy of geolocalization (for all measurement points with geolocalization) in function of time, since the first second of measurement.

The ‘accuracy’ data collected by the NoiseCapture application allow one to associate a location accuracy (in meters) to each measurement. This value is obtained using the getAccuracy() function in Android [44], meaning that there is a 68% probability that the true location is inside the circle (with a radius equal to the value of the ‘accuracy’) centered at the corresponding location. The analysis of this parameter shows that the median value of ‘accuracy’ is around 8 m. It should also be mentioned that it is possible to find some measurement points with non-realistic accuracy values (such as $1.1\times \;10^{5}$ m) that may due to a wrong technical implementation of the GPS algorithm in the smartphone. For points with geolocalization, Table 11 shows that most of accuracy are under 25 m (42,313,601 points, 86.5%), which can be considered as a relevant accuracy for noise studies [45], and 35,184,828 (71.9%), 18,069,523 (36.9%), and 420,150 (0.8%) under 15 m, 5 m, and 1 m, respectively. Finally, one can observe from Figure 8 that the accuracy tends to increase (i.e., the accuracy value decrease) when the measurement duration increases. This is due to the fact that a sufficient duration may be required for the GPS receiver within the smartphone to detect GPS satellites, and then to obtain the best accuracy of location. It suggests that a NoiseCapture user should wait few seconds after starting the application (for example, ref. [14] mentions a duration of 4 seconds), before performing a measurement, in order to obtain the best geolocalization.

Last, it must be mentioned that the function getAccuracy() returns the value 0.0 when the smartphone is not able to obtain a value for the accuracy. This should not be consider as an accuracy value of 0 m.

#### 3.3.3. Speed

The NoiseCapture data set contains information about the ‘speed’ value, which represents the speed (in meter per second) measured by the smartphone GPS at the time of the measurement point. Table 12 shows that 38.5% of tracks (65.4% of the measurement points) have a speed equal to 0. According to the Android documentation for the getSpeed() function [46], a null value is returned when the location does not have a speed; it does not mean that the speed is equal to zero, but that it is not possible to evaluate its value, even when the measurement is geolocalized. Note also that it could be interesting to also collect the estimated speed accuracy using the Android function getSpeedAccuracyMetersPerSecond() in a future release of the application.

When the speed value is different greater than 0, it means that the measurement point is moving, but it may be difficult to determine in a simple way the transportation mode that is used (walking/running, bicycle, light vehicle, public transportation, etc.), with the only knowledge of the speed value, as the speed ranges corresponding to each transportation mode may overlap [47]. Assuming people walking at a speed ranging from 0.5 km/h to 5 km/h (0.14 to 1.4 m/s, respectively), one can consider that around 14.4% of the measurements are realized during walking. One can also find speed values that correspond very clearly to measurements carried out in fast mode of transportation, including air transportation of the order of 280 m/s.

Additional analysis of the ‘speed’ information also shows several anomalies, such as negative values (26,304 measurement points, 0.04%), with the ‘−2’ (26,257 meas.) or ‘−1’ (14 meas.) values; such values may probably have a signification, but this information is missing in the Android documentation. Other negative values (33 meas) are in the range $\left[-1,0\right]$ and may be due to numerical accuracy.

### 3.4. Temporal Characteristics of Measurements

#### 3.4.1. Measurement Timestamp

As already mentioned, the analysis developed here concerns an extraction of the database, as the official launch of the application over a period of 3 years, from 29 August 2017 to 28 August 2020 (considering all versions of the application since number 28). At the time of a measurement, the beginning of a ‘track’ is defined by the field record_UTC (given by the smartphone) and each ‘point’ of a ‘track’ is defined by the field time_location (given by the GPS).

The analysis of the entire database (i.e., between the date of the track record_UTC and the date of the first point time_location in the corresponding track), shows some measurements that are visibly incorrectly time-stamped; this corresponds to points without geolocalization (defined with the time_location=’1970-01-01’ by default). One can also observe measurements (21,897 tracks, 849,128 points) with a time shift of several hours (Table 13), but it represents less than 2% of the total number of tracks. Last, it is also possible that some users use date and location metadata scrambling tools on their smartphone to avoid tracking. The number of tracks/points concerned being however very low, one can imagine that the database analyzed here is little or not at all concerned by this type of error.

**Figure 9:**
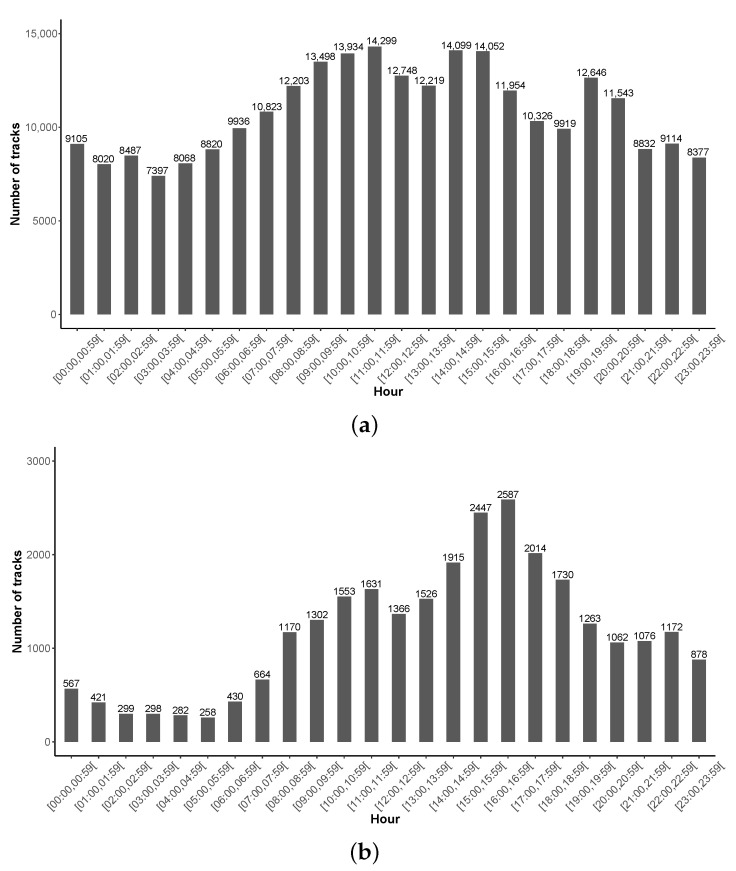
Distribution of tracks per hour of the day, for the entire database and for the data collected in France only. (**a**) Distribution of tracks per hour of the day, for the whole database. (**b**) Distribution of tracks per hour of the day for data collected in France only.

Figure 9 illustrates the distribution of the tracks in function of the hour of a day. For the entire database (Figure 9a), one can observe a moderate variation from one hour to another, which can be explained by the fact that measurements are collected in all the time zones simultaneously (assuming that measurements are done all over the word simultaneously). When focusing on the data collected in France only, Figure 9b shows, as expected, a small number of measurements during night and early morning and more measurements during day and afternoon.

**Figure 10:**
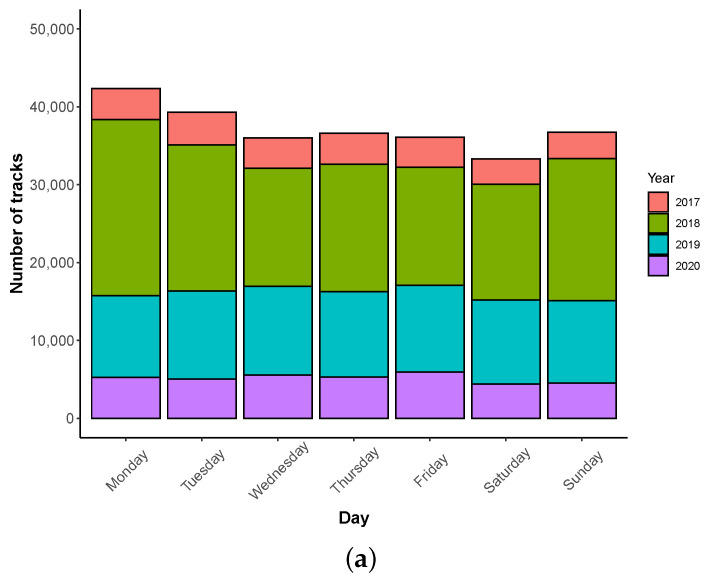

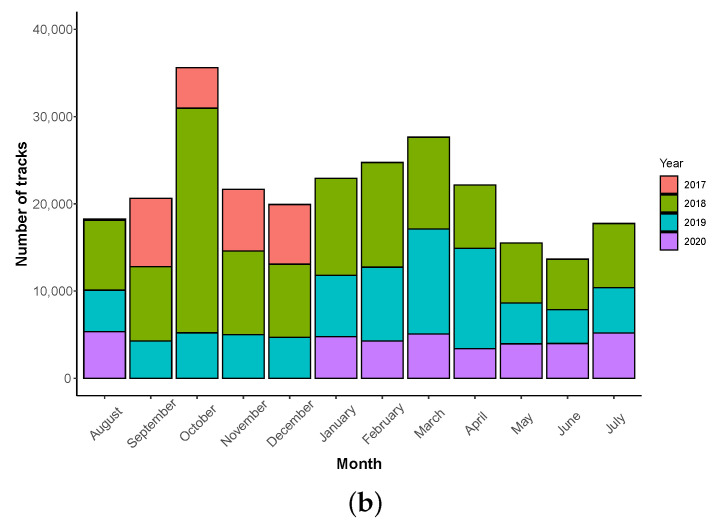
Distribution of track measurements in function of the day and the month, over the 3 years of the collected data. (**a**) Distribution of tracks per day of the week. (**b**) Distribution of tracks per month.

In addition, Figure 10 illustrates a small variation from one day/month to another, except for ‘October’ with more tracks for the year 2018, due to an unusual and large amount of data (8470 tracks) collected on 8–9 October 2018, by few users only, localized in Peru (6326 tracks, 78,789 points). This can be due to a specific event.

#### 3.4.2. Measurement Duration

**Figure 11:**
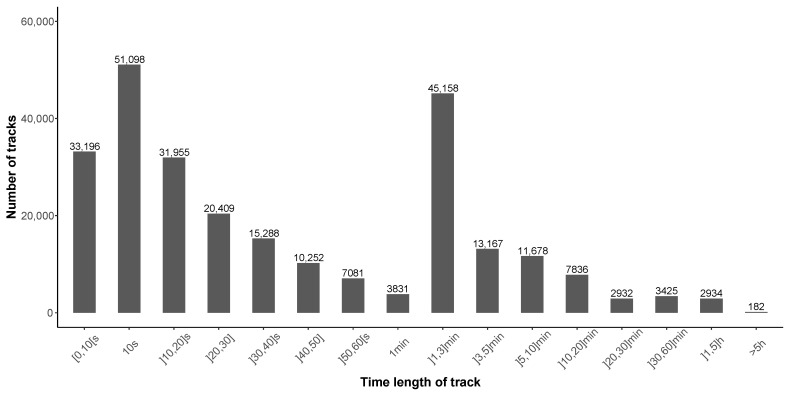
Distribution of tracks in function of the duration of the measurement (‘time_length’).

The ‘time_length’ data (in second) are defined from the start of the measurement during a track, until the user ends the measurement. Figure 11 shows that most measurements are done with a track duration around 1–20 s (44.6%) and 1–3 min (17.3%). Only 6.6% of tracks have duration greater than 10 min. The 10 s duration corresponds to a large part of the measurement (51,098 tracks 19.6%); this is due to the fact that user can used a predefined duration, which is fixed to 10 s by default. One can note that measurement duration between 1 and 3 min has also as strong presence. Table 14 shows that only small percentage of user collecting tracks with long period move along the track (≤6.45%, when considering a minimum speed value of 0.5 m/s).

An issue can be observed regarding the total number of measuring points (59,685,328), which is greater than the sum of all the measurement track duration (∑time_length = 59,684,657), with a difference of 671 s (i.e., points). Analyzing this issue, one can observe that 82 measurement tracks (made by different users, with different devices, using different application release) have a number of points (i.e., seconds) that are not equal to time_length. In addition, one can mentioned that few points have been removed from their corresponding tracks. For now, the exact reason of such anomalies is still not defined, but may be due to an unusual behavior of the application or to numerical inaccuracies.

### 3.5. Smartphone Acoustic Calibration

The relevance of the collected acoustic measurements is largely based on the measurement protocol applied by the contributor as well as on the metrological quality of the smartphone. At this stage, concerning the first point, it is expected that the contributor follows the recommendations available in the application. No other information is included in the collected data in order to analyze if this measurement protocol is well followed (excepted for the calibration); this point will be discussed in Section 4. The second point has given rise to numerous studies in the literature, as it is a critical element.

It is indeed hoped that the user calibrates his/her smartphone before collecting measurements. Numerous studies, among them recent ones [48,49,50], have shown the need to make a correction on the values measured by smartphones, in order to get closer to those that would have been measured with a reference device, such as a sound level meter. However, from a statistical point of view, this condition is not as critical, since it can be expected that due to a large number of measurements collected by different smartphones, the results may statistically converge to the expected values. This hypothesis seems to be confirmed by Murphy and King, showing, that in the absence of calibration, the sound levels measured on average by a wide variety of smartphones are very close to the expected value, but at the expense of a large standard deviation. Acoustic calibration can however reduce the standard deviation.

In the NoiseCapture application, the calibration procedure consists in evaluating a correction (or a calibration gain, i.e., the ‘calibration_gain’ value) that will be applied on the input temporal signal before the postprocessing of all noise indicators, assuming a linear relationship both in frequency and in amplitude, which is of course questionable for some smartphones. Within NoiseCapture, several calibration methods are proposed and defined by the field ‘calibration_method’ in the database:

- The most relevant solution (’Calibrator’ method) consists in using an acoustic calibrator, according to the classical rules for acoustic measurement. This solution requires an external microphone, connected to the smartphone, with a diameter compatible with the use of an acoustic calibrator. Note that using an external microphone can also improve the measurement accuracy in comparison with the internal microphone [51]; thus, this solution must be promoted to contributors. The ‘calibration_gain’ is then determined for a reference frequency and for a reference level (for example 94 dB@1 kHz) and applied, during measurements, to the entire temporal signal before processing;
- Another method (’Reference’ method) is used to correct the sound level measured (overall or for a given frequency) by the smartphone using another measuring device (i.e., using a visual comparison), considered as a reference. The value of the gain ‘calibration_gain’ is obtained from an ambient noise measurement.
- A third method (’CalibratedSmartPhone’ method) is used to calibrate one or more smartphones simultaneously, using an already calibrated smartphone as a reference. The procedure is fully automatic, controlled by the reference smartphone, and is based either on the measurement of the ambient noise or a pink noise generated by the reference smartphone.
- Finally, a more recent method (’Traffic’ method) is based on the measurement of several pass-by of light road vehicles, which, by comparison with a statistical model of noise emission, makes it possible to estimate the correction to be made to make the measurement coincide with the expected statistical value [52].

The user can also directly change the value of the calibration gain in the application settings at any time. The default value of ‘calibration_gain’ is set to 0 dB as long as no calibration method has been applied, or as long as the user has not directly changed this setting. A change of this parameter will be considered as a ‘ManualSetting’ for the ‘calibration_method’ field.

The choice of the method is defined by the field ‘calibration_method’, but only since version N°49 (17 February 2020). If, since the launch of the application, several calibration methods were already available, the information on the choice of the applied method was not known and only the value of ‘calibration_gain’ was actually uploaded to the remote server. For database consistency reasons, all data collected using versions prior to version 49 of the application, the choice ‘None’ is affected to ‘calibration_method’, although a calibration method may have been used. However, since version 49, the choice ‘None’ is only affected when no calibration is performed.

Table 15 shows the distribution of the collected tracks according to the calibration method. As indicated above, the field that defines the choice of the calibration method, is only available since release 49. By analyzing the data collected before release 49, one can observe that 62,731 of the 241,532 collected tracks, i.e., ~26%, have a calibration value different from 0, meaning that the corresponding users have probably applied either a calibration method or a manual change of the calibration gain in the settings of the application.

From release 49, we can see that 7.9% of the tracks have been made by smartphones that have been calibrated (92.1% are defined by ‘None’ for the ‘calibration_method’), but for about half of them, the manual method has been applied. For these tracks, it is therefore difficult to determine how the value of the calibration gain has been evaluated. Among the other calibration methods, the one using the automatic procedure between smartphones is the most used, followed by methods using a reference and an acoustic calibrator. The traffic calibration method is the most recent and has generated little data so far. In the future, it will be important to highlight this last method, which is the only one that is able to calibrate a smartphone without the need for an external device, while offering sufficient accuracy.

**Figure 12:**
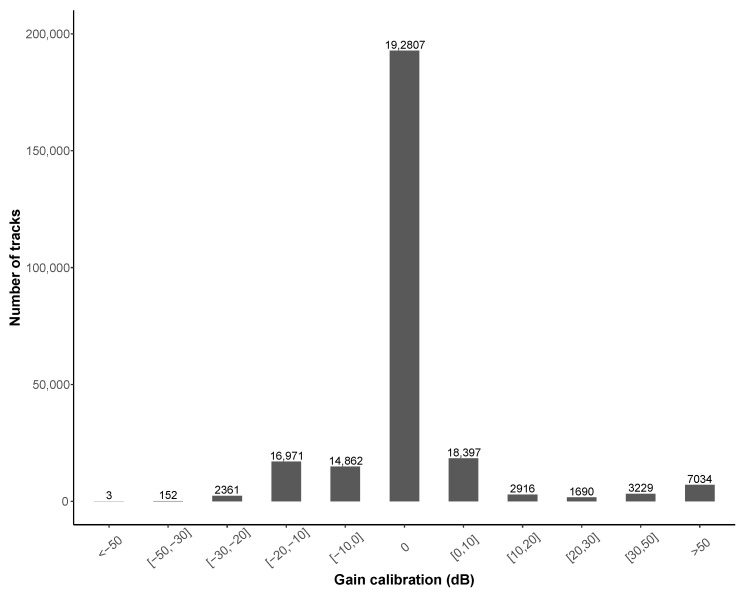
Number of tracks in function of ‘gain_calibration’ value.

The application of a calibration method is not enough to justify the quality of the measurements. The obtained value of the calibration gain (field ‘gain_calibration’) is also a very important information. Figure 12 illustrates the distribution of tracks according to this value and brings some comments. The presence of abnormally high (in absolute value), even extreme and aberrant values shows either a bad use of the calibration methods or a technical problem. The number of tracks collected with a calibration gain of zero (default value) globally reflects a lack of calibration, as it is unlikely that a smartphone is calibrated by default. Finally, we can see that 86.8% of the collected tracks have a calibration value between −10 and +10 dB, which seems rather realistic, but does not bring any certainty on the quality of the measurement.

Table 16 completes this first analysis by showing the distribution of calibration gain values according to the method (data collected since release 49). As expected, it can be observed that when a calibration method considered as ‘robust’ is applied (’CalibratedSmartPhone’, ‘Calibrator’ and ‘Traffic’), the gain is different from 0 dB, except for the ‘Reference’ method. There is also slightly less disparity in the gain values when a calibration method is applied. One can also observe that for the ‘Calibrator’ method, 90.7% of the calibration gain values are greater than 10 dB, which shows a different behavior than other methods. This may be a misunderstanding of the method, with some users attempting to calibrate their smartphone without a reference device.

The analysis of these calibration data, for example, in relation to the type of device and user profile, is in itself a separate study. The creation of a ‘validated’ database of calibration values for each smartphone model, for example, is an interesting prospect. However, this perspective study is beyond the scope of the present article, which, at this stage, only aims to present the data collected and their limits of use.

### 3.6. NoiseCapture Parties

As mentioned above, a NoiseCapture Party is a special event organized by a given organization, aiming to carry out measurements, generally over a limited time and a spatial area, for educational, scientific dissemination, or research purposes. The advantage of these events lies in the fact that the measurements are generally well ‘controlled’, and most of the smartphones have been previously calibrated. It can thus be considered that the collected measurements have a better quality compared to the other measurements in the database.

The list of all NoiseCapture Parties are given in Table 17, with the number of considered smartphones and the total of collected tracks and points. As expected, the ratio of calibrated smartphones is greater for NoiseCapture Parties. The total number of collected tracks and points represents 1.2% and 0.6% of all data in the database.

It must be specified that other similar events could have been organized, without having given rise to the creation of a specific tag, and without having informed the developers of the application. For example, this is the case for several recently published research works [53,54,55,56,57].

### 3.7. Soundscape Description

NoiseCapture allows users to complete the acoustic measurement with information about his/her own perception of the sound environment, using ‘tags’ (field   ‘noisecapture_track_tag’) for describing the noise environment and the noise source along the track. In addition, they can give an information of ‘pleasantness’ (field   ‘pleasantness’) by selecting a value (0 for ‘unpleasant’, 25, 50, 75, 100 for ‘pleasant’); this field may be empty if no value is selecting (default value).

The analysis of the tags can be particularly interesting to distinguish the measurement conditions; indeed in certain conditions, such as rainy or windy weather, the acoustic measurement may be distorted, and it is therefore interesting to have such information before analyzing the acoustic indicators. The information about the indoor/outdoor measurement is also interesting for people who would like to use the data to characterize indoor or outdoor sound environment, specifically. Last, the knowledge of the sound sources that are perceived and the evaluation of the pleasantness are also interesting data for researchers that study the notion of soundscape.

Both ‘Pleasantness’ and ‘Tags’ are supplementary info. Their use add beneficial information about the sound environment. Ideally, this information should be systematically provided by the contributors. However, Table 18 shows that only 17.5% of the tracks have both information, while 48.7% do not have any and 33.8% have either one of them (mainly the pleasantness with 30.2%). An independence test showed that there is a dependency between using both ‘Pleasantness’ and ‘Tags’: a participant using tag will use pleasantness more often.

#### 3.7.1. Pleasantness

**Figure 13:**
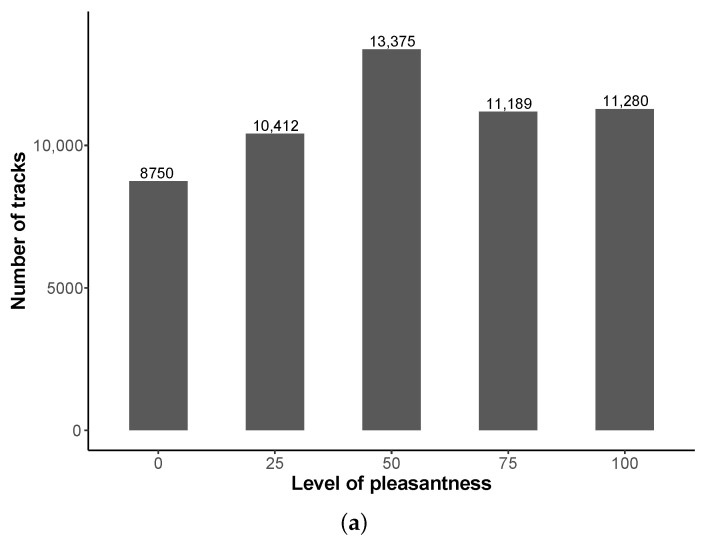

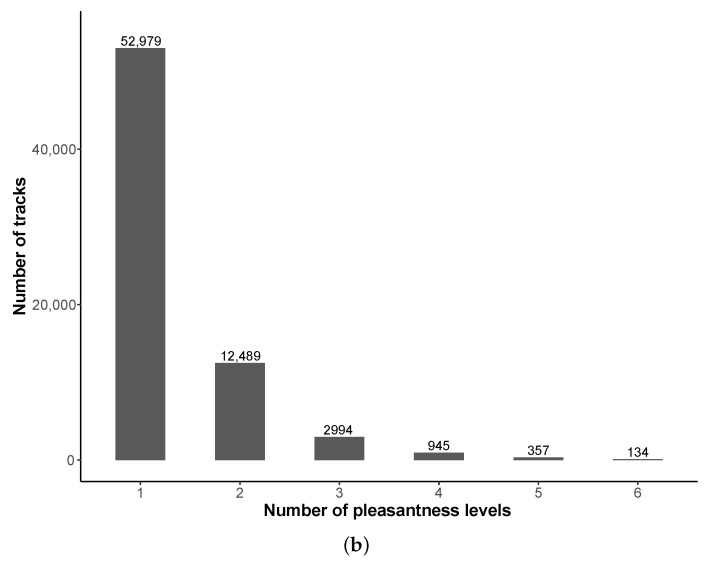
User evaluation of pleasantness on a track: (**a**) Distribution of pleasantness values on tracks. (**b**) Distribution of number of pleasantness levels used by contributors.

Figure 13a shows that most of tracks (205,416 (78.9%), equivalent to 46,623,131 points (78.1%)) are not associated with a value of pleasantness, meaning that the default empty value is not modified. Excepted for the level ‘50’, all other values are used quite uniformly. The over-representation of the level value ‘50’ can be explained by three possible reasons:

1. for the most part, users cannot judge the quality of the sound environment in a clear-cut way;
2. by default, the selection cursor is positioned on the value ‘50’ that can influence the user;
3. the user may be tempted to select the cursor, without however wanting to make a decision. Once the cursor is activated, it is no longer possible to go back and a value will be automatically validated.

The two last hypothesis can introduce a bias, suggesting that a more suitable selection mode should be proposed in a future release of the application.

The behavior of a contributor can also be analyzed in terms of his propensity to use all the possible values of the pleasantness scale (Figure 13b). This analysis shows that for 52,979 users, only 1 level is used; however, in detail, for 43,764 of them (i.e., 82.6% of 52,979), the ‘NULL’ value is used; 12,489 used 2 levels, etc. Few users therefore use the pleasantness scale, and even fewer use these different levels of the scale. In a future evolution of the application, it could be interesting to ‘motivate’ users to provide information, for example by ‘forcing’ them to give an answer, including a ‘don’t say’ answer.

#### 3.7.2. Tags

In addition to the perception of their sound environment, users also have the possibility to specify the measurement conditions (4 tags) and the nature of the perceived sound sources (14 tags in four categories: human activity, transportation, natural, and mechanical activity). Table 19 gives a description of the tag fields in the database (‘pk_tag’ and ‘tag_name’) as well as the corresponding English description within the NoiseCapture application (see Figure 1). The list of ‘pk_tag’ is not continuous, some missing numbers correspond to tags that are no longer used since the first official release of the application.

**Figure 14:**
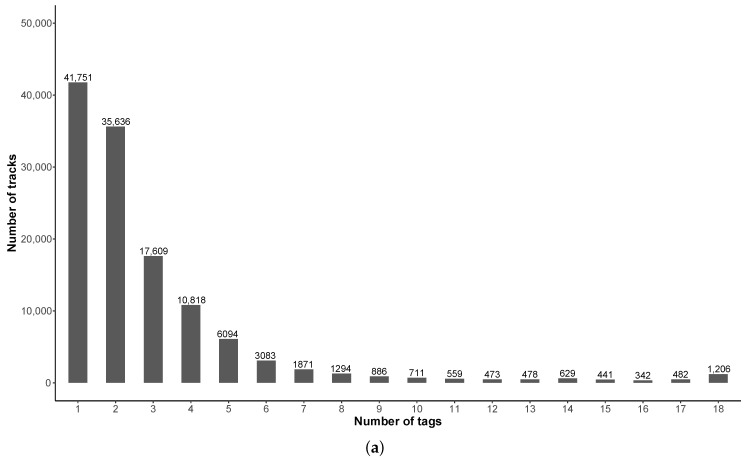

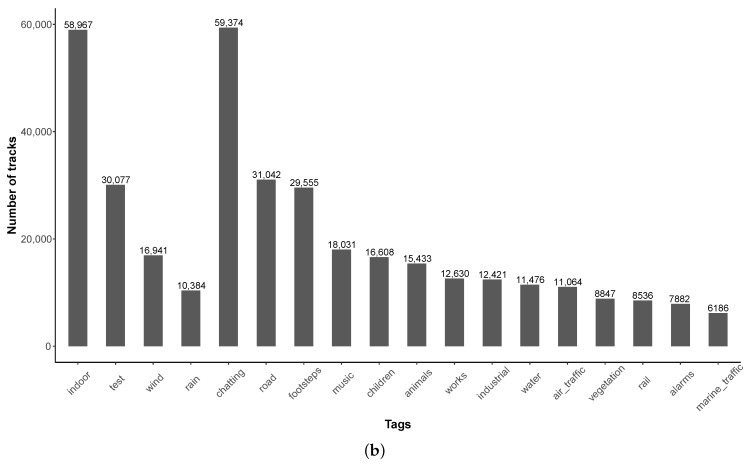
Use of soundscape tags by contributors. (**a**) Number of tags simultaneously used in a track. (**b**) Tags name.

Figure 14a shows the number of tags that are simultaneously used to describe a track. In about half of the collected tracks (124,363, 47.7%), the contributors do not use any tag to describe the measure. This is better than for the pleasantness evaluation.

Nearly 30% of the tracks contain 1 or 2 tags: when considering 1 tag only, 17,094 tracks (40.9%) are defined by an environment tag (‘test’ or ‘Indoor’); when considering 2 tags, 2061 tracks are defined by 2 environment tags, 9260 tracks by 2 source tags and 24,315 tracks by a combination of two types of tag. One can also note that a number of tracks simultaneously contains a large number of tags, even the 18 possible tags, which is not realistic. We can assume that the corresponding tracks are test measurements, but they do not necessary mention the ‘test’ tag. Figure 14b shows that this ‘test’ tag is used in 30,077 of the collected tracks, which is important. An analysis of the database, for the purpose of studying sound environments, will necessarily exclude the collected data with this tag.

The other interesting aspect is that the ‘Indoor’ tag is present in 58,967 of the tracks collected, which represents an interesting quantity for the study of sound environments in closed spaces (building, transportation), even though the initial objective of the application was to study outdoor environments. Note also, that the tags ‘Indoor’ and ‘Test’ are not independent, meaning that both tags can be used together.

Among the sound sources mentioned by the contributors, ‘voice’, ‘footsteps’, and ‘road’ are present, which is consistent with a contributor who collects measurements closed to road infrastructures, while walking and talking. Again, it is obvious that the analysis of these tags and their occurrence can provide interesting information on the perception of sound environments. However, this is beyond the scope of this article.

### 3.8. Noise Indicators

The purpose of the NoiseCapture application is based on the measurement of acoustic indicators for the analysis of sound environments. The data that are present in the NoiseCapture database concern the equivalent sound level $L_{A,\mathrm{eq}}$ on a track, as well as the spectrum and sound level at each point of the track, measured every second. The postprocessing of these data can, in a second step, give access to percentile indicators (such as $L_{A10}$ or $L_{A50}$) or to sound level distributions, for example. In the following, the analysis is restricted to the data as such, and not to the sound environments.

First of all, it should be remembered that in terms of acoustic measurement, smartphone manufacturers under the Android OS must respect a number of recommendations defined in the Android Compatibility Definition [38]. In particular, they should offer (1) an audio capture with approximately flat amplitude and frequency characteristics of ±3 dB from 100 Hz to 4000 Hz, (2) an input sensitivity such that a 90 dB Sound Power Level (SPL) at 1000 Hz gives an RMS value of 2500 for 16-bit samples, and (3) a linear change of the amplitude over a range of at least 30 dB from −18 dB to +12 dB relatively to 90 dB SPL at the microphone. Some smartphones may offer superior features, but it is expected that all smartphones meet the minimum requirements.

**Figure 15:**
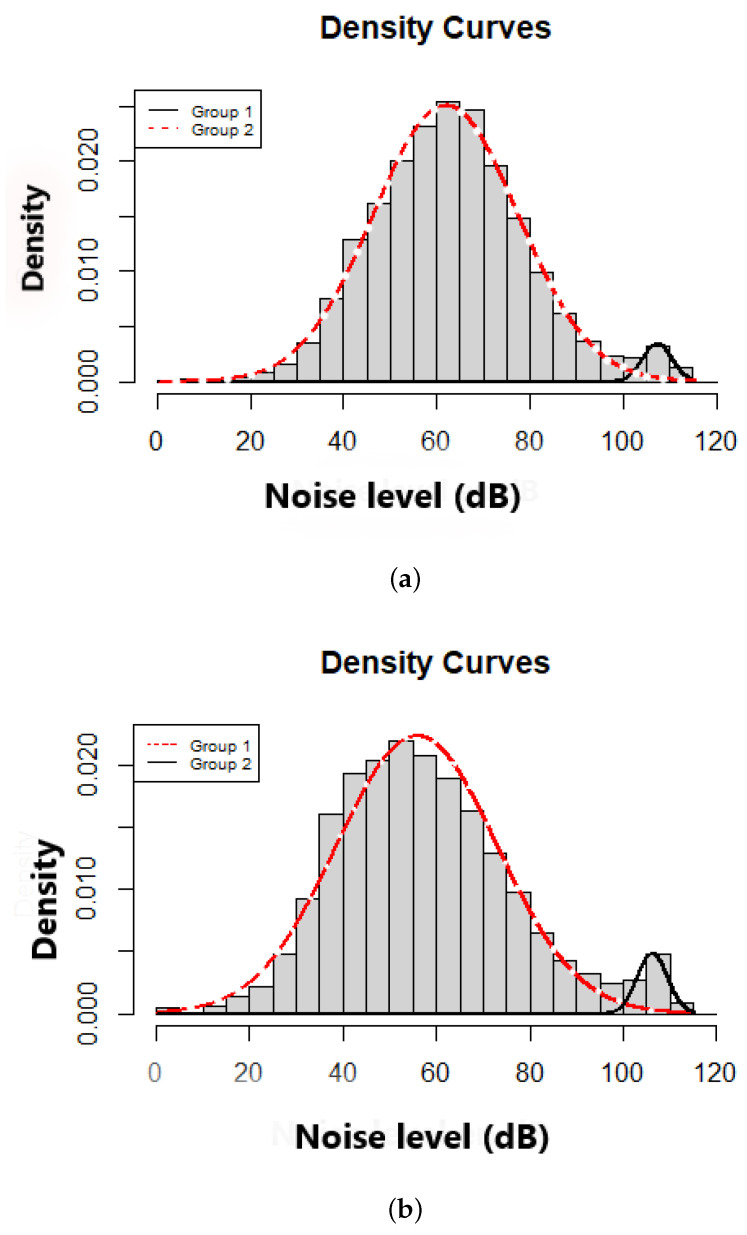
Distribution of noise levels (**a**) on the 260,422 tracks and (**b**) on the 59,685,328 points.

Figure 15 shows the range of $L_{A,\mathrm{eq}}$ values measured along the tracks and at each point of a track. While not visible in this figure, one can observe data with very low sound levels (a few decibels, even negative ones), which seems physically both unrealistic in a real environment, but also *a priori* outside the measurement capabilities of a smartphone. On the other hand, the highest levels are of the order of 125 dB, which is not unrealistic but, nevertheless, unlikely in a normal environment.

In details, Figure 15a shows that the noise levels measured on tracks can be represented as a mixture of 2 normal distributions (noted N(mean,standarddeviation)), a first group $X_{1}$ defined as ∼N(107.4,3.1) and a second group $X_{2}$ defined as ∼N(62.0,15.5). These normal distribution are also respectively defined by a ‘gain_calibration’ of mean 73 and 0 dB (median 80 and 0 dB). Figure 15b shows similar results for the noise levels at the measurement points, as a mixture of 2 normal distributions, ∼N(106.3,3.2) and ∼N(50.9,17.2) with similar statistical values for the ‘gain_calibration’. For highest sound levels, it is quite evident that the calibration was not performed correctly.

This simple study shows that the range of variation of the measured sound levels is abnormally wide, the absence of calibration or a bad calibration being a probable cause.

## 4. Discussion and Future Developments

### 4.1. Synthesis

The analysis of the data collected during the first 3 years of operation since the launch of the application clearly shows that the information may be made of anomalies and uncertainties. Quantifying and reducing some of these biases is possible, either by a better knowledge and control of the user’s behavior and of the context of measurement or by improving the smartphone application.

Table 20 already proposes at this stage some simple modifications to implement within the application, mainly by checking some settings (verification of the smartphone date/time, activation of the geolocalization, user profile update, change of the ‘Pleasantness’ selection mode). Most critical aspects concern the lack of a good geolocalization and bias in the noise level measurements mainly due to a wrong or a lack of smartphone calibration. These two subjects are specifically discussed in Section 4.2 and Section 4.3. In addition, a better knowledge of the context of the measurement could also judiciously complete the collected data, or even replace certain user actions, such as the use of tags. Some suggestions will be proposed in Section 4.4. Last, increasing trust in the data also means increasing trust in the users. The animation of the community of contributors is another essential challenge. This is discussed in Section 4.5.

### 4.2. Increasing Localization Accuracy

In the current release of the application, localization is performed in an elementary way, and it was realized afterwards that it may not be sufficient depending of the objective of the use of data. With a view to improve the performance of the application (and therefore the quality of the data produced), the quality of GPS localization is a point on which the user must be made aware. In the application, this can for example take the form of recommendations to improve the quality of GPS location, such as activating the ‘High accuracy’ mode in the Android settings, re-calibrating the GPS *via* the use of a third party application, or activating additional localization functions *via* WiFi, Bluetooth and mobile networks.

### 4.3. Building a Smartphone Calibration Database

Some authors have rightly proposed to provide contributors with a database to calibrate smartphones, in order to limit bias during an acoustic measurement. Building such a database can be tedious because of the large number of smartphone models present on the market simultaneously, as well as their very rapid evolution. However, the NoiseCapture experimentation has opened new perspectives. Indeed, the analysis carried out in the present article shows that a large part of the contributions come from a limited number of manufacturers and models (three manufacturers account for about 35.2% of the models and nearly two thirds of the tracks; 15 models only have 15.9% of tracks). This information would limit the number of calibrations to be performed in the laboratory to build a calibration database. The other perspective would be to use the calibration data proposed by the contributors for their smartphone. The quality of this calibration can however be discussed, except for contributors performing their calibration during a NoiseCapture Party type event.

### 4.4. Collecting Information about the Context Awareness

From the very beginning of the application creation, the kind of the information sent back to the remote server was deliberately restricted to what was strictly necessary, so that it would not be considered as invasive. The study of the data collected over 3 years nevertheless shows that their use in a better controlled scientific approach would require additional information.

In particular, information on the context of the measurement, such as wind detection, activity recognition, transportation mode detection, how the smartphone is used during measurements, and place recognition [47,58,59,60,61,62] could be useful. The use of information provided by other smartphone sensors (accelerometer, orientation, brightness sensor, and proximity sensor) could also provide information on the process of the measurement [14,17]. Note also that, as mentioned in [47], specific functions are already available in Android API to identify some user activities [63], which could be a first attempt to obtain new information. To a lesser extent, it may also be interesting to collect the speed accuracy (adding a new data ‘speed_accuracy’), since this value is also available in Android API.

Providing that smartphones have sufficient resources, the integration of sound source identification algorithms can also give interesting additional information, and can advantageously replace the use of tags [64]. Otherwise, it should also be possible to include such identification as a postprocessing on the remote server, for example, by using the collected 1 s spectra. All these development perspectives must nevertheless be integrated in the total respect of the privacy of the contributors [65], in particular, in the respect of laws in specific Regions/Countries, such as the General Data Protection Regulation (GDPR) in Europe [66].

### 4.5. Increasing and Animating the Community of Contributors

The participatory approach is of course the main originality of the application, allowing one to considerably multiply the number of measurement points, with a large variability in time and space. Like any participatory approach, the main challenge is to maintain the initial interest of the contributor to support a research project or make their individual contribution a major social issue (i.e., noise pollution) [67], beyond a time of discovery and a few measurements. The analysis of installs/uninstalls detailed in Section 2.4 shows indeed a tendency to a negative imbalance between installs and uninstalls of the application, which suggests that it is important to propose a solution to better retain users. Moreover, the analysis of the contributors behavior in Section 3.2.3, shows that there are finally few active contributors (half of the contributors made only one measurement, mostly to test the application), and that almost half of the contributions do not exceed 20 s (38.5%, Table 8). It is therefore require to develop strategies that allow for the development of a community of very active contributors.

This must be achieved by enhancing the application in order to motivate users to regularly produce measures, for example by adding reminder notifications to contributors or by developing a more playful aspect (creation of pseudonyms, setting up a challenge or a serious game based application such as noise battle or noise quest [68], creating badges…). If the target is more oriented towards a community of professionals (i.e., the initial target), the animation of the community can be more distributed, by calling upon ‘ambassadors’ (teachers, student researchers, technical agents of communities, government services…) who will see a particular interest in organizing, for example, NoiseCapture Parties. As also mentioned by others authors [48,67,69,70], the advantage of organizing ‘controlled’ events lies in the possibility of training users to carry out measurements using a validated protocol, particularly from the point of view of the calibration of smartphones, which would increase ‘confidence’ in the measurements. Once trained, users could in turn train other users, increasing the ‘trusted’ community.

Implementing a serious game type application, or increasing interactions with contributors, should also encourage the contributor to take measurements in specific spaces and at specific times. As considered by the authors of [14], this would make it possible to compensate for a lack of measurements in certain places or at certain times.

The analyzing of the geolocalization of the measurement points also showed a inhomogeneity in the diffusion of the application throughout the world, mainly due to the initial choice of the development platform (Android) and the associated application stores (on Google Play only). If one can observed the very wide use of the application throughout the world offers (currently, the application has been used in 204 different countries), this analysis shows the need to disseminate the application even more widely in order to acquire data in some countries with large populations. This could offer a wealth of data that is particularly interesting from the point of view of evaluating sound environments in countries with very different cultures and environments.

## 5. Conclusions

The use of a crowd-sourcing type approach offers interesting perspectives in the analysis of sound environments, in particular because of the spatial extent and the temporal dynamics that the data collected can provide. The involvement of citizens in a collaborative approach also brings another dimension to scientific research on the subject. The initial and legitimate fears about the relevance of using such data in an environmental approach (evaluation of public policies to reduce noise nuisance, effects of noise on health, perception of noise environments) are being allayed. Studies have indeed shown the relevance of this type of approach [67,69], while underlining some important points, for example, users proactivity, critical mass of contributors, increasing of measurement accuracy or the need of organizing collective sessions of noise sensing, etc.

The development of the NoiseCapture application is fully in line with this alternative approach. Compared to similar approaches, however, the NoiseCapture approach offers a completely open source platform, ensuring total transparency on the methods of collecting and processing data, and giving the possibility to everyone to freely use the data. The sustainability of the approach was also considered, by making effort to ensure the functioning of the project over time. These specificities are certainly the reasons for the success of the approach, whether it be with many communities.

**Figure 16:**
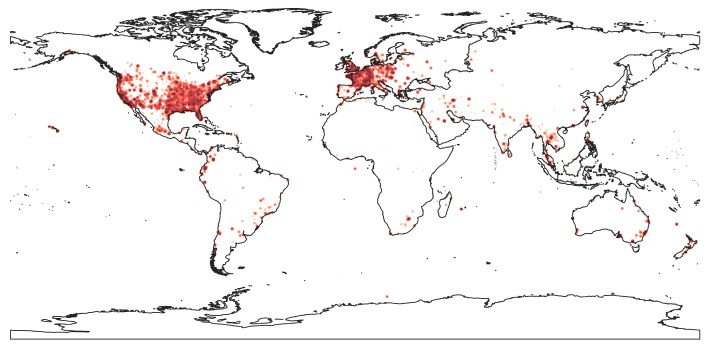
‘Heatmap’ representation of the NoiseCapture data collected around the world.

Since the launch of the application on 29 August 2017, the amount of data collected is considerable. After 3 years of operation, thanks to the participation of 74,082 contributors, the database has accumulated 260,422 tracks and 59,685,328 one-second measurement points, spread over 204 different countries (Figure 16). To our knowledge, there is no other similar experimentation.

Although the amount of data collected is considerable, any exploitation of the database for applications related to the study of sound environment requires a perfect understanding of the data, in order to limit bias in the analysis. The objective of this article was therefore to review all the data collected (nature, content, limits, etc.) and to identify specific behavior linked to the use of the application (Section 3). This analysis now provides a precise framework for the further exploitation of the data. In view of the very large amount of data collected, it is however clear that depending on the nature of the expected analysis, a large part of the data cannot be used, either because it does not present any interest for the corresponding analysis, or due to a lack of completeness and accuracy. As discussed in Section 4, in our opinion, enhancing/controlling the quality of the data and of the measurement conditions constitute two major developments for improving the database. The other major perspective consists in the animation of the contributors community to increase confidence in the data.

**Figure 17:**
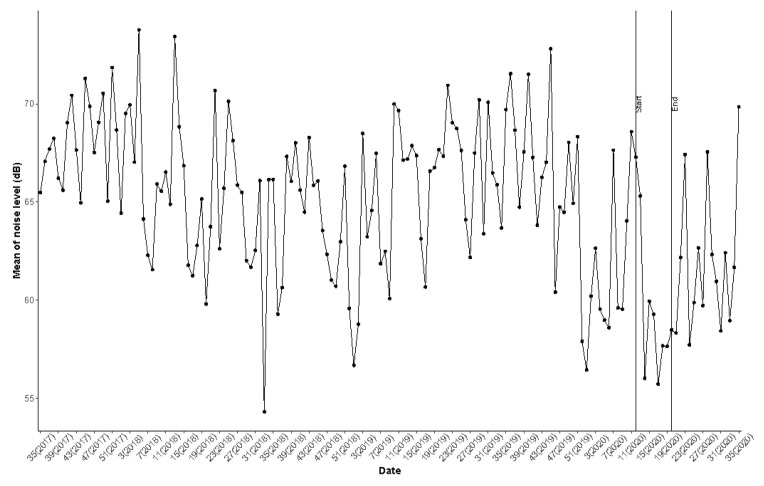
Distribution of mean ‘noise_level’ in France by week accompanied with 2 verticals lines that represent the start and end of the first lockdown.

Thus, as soon as attention is paid to the inherent limits of the collected data, the exploitation of this database offers very interesting perspectives on the characterization of sound environments. Any relevant analysis could be useful for communities to assess the noise environment of their territory, and usefully complement regulatory requirements, such as the 2002/49/CE Directive, in Europe, relating to the assessment and management of environmental noise. As an example, a very simple analysis of the sound levels collected in France shows, without any particular treatment, an overall decrease in sound levels during the periods of lockdown related to COVID (Figure 17).

Beyond the exploitation of the database, one can also mention that the use of the NoiseCapture application with a dedicated use of the collected data (i.e., without using the NoiseCapture database, but using only the data export capabilities of the application) can be an interesting tool for scientific purposes [53,54,55,56,57].

## Figures and Tables

**Table 1:** NoiseCapture application releases. Each new version, defined by a version release (for example ‘51’) and a version number (for example ‘1.2.15’) proposes changes (bug corrections, user interface enhancement, etc.). The reader can refer to the detailed list of fixes in each (pre-)release from the GitHub source code management platform [27]. The changes made on the data export, from the smartphone to the data server (adding new data, patches, etc.), are detailed in the history of the source code file MeasurementExport.java [28]. The date of publication of the application on Google Play is also provided for information (corresponding official public release are indicated in bold with symbol *).

Release (Number)	Source Code Publication	STATUS	Application Publication	Comments
51 * (1.2.15)	3 July 2020	Release	7 July 2020	Fix automated measurement upload
49 (1.2.13)	17 February 2020	Pre-release		Add calibration method using road traffic
				Add ‘calibration_method’
45 * (1.2.9)	27 March 2019	Release	26 March 2019	Calibration in $L_{A,\mathrm{eq}}$ instead of $L_{\mathrm{eq}}$
43 * (1.2.7)	16 November 2018	Release	16 November 2018	Minor changes
35 * (1.1.3)		Release	20 April 2018	Minor changes
34 * (1.1.2)		Release	29 January 2018	Minor changes
33 * (1.1.0)	23 November 2017	Pre-release	24 November 2017	Ability to use a calibrated sMarchtphone
				to automatically calibrate other sMarchtphone(s)
32 * (1.0.4)		Release	6 November 2017	Minor changes
31 * (1.0.3)		Release	6 October 2017	Minor changes
30 (1.0.2)	18 September 2017	Pre-release		Add NoiseCapture Party functionalities
29 * (1.0.1)		Release	31 August 2017	Minor changes
28 * (1.0.0)	23 August 2017	Release	29 August 2017	Official first release
				Add ‘user_profile’

**Table 2:** Distribution of collected data per release, from 29 August 2017 to 28 August 2020. The number of collected data during NoiseCapture Parties (see Section 3.6) is also indicated, as well as in terms of percentage of the total number of data. Releases in bold with symbol * correspond to public releases on Google Play. Note that the total number of contributors in this table (74,082) does not correspond to the total of unique contributors, as a contributor may have use several release of the application.

Release	Contributors		Tracks		Points	
	Total	Party	Total	Party	Total	Party
28 *	26	–	354	–	46,268	–
29 *	2705	–	8991	–	1,588,156	–
30	35	7 (20.0%)	416	133 (32.0%)	140,627	11,523 (8.2%)
31 *	1432	2 (0.1%)	4426	6 (0.1%)	957,920	770 (0.1%)
32 *	1553	3 (0.1%)	4746	9 (0.2%)	847,093	1556 (0.2%)
33 *	5442	4 (0.1%)	19,225	52 (0.3%)	3,530,349	8819 (0.2%)
34 *	9053	13 (0.1%)	28,607	67 (0.2%)	6,121,154	18,793 (0.3%)
35 *	15,734	67 (0.4%)	68,911	921 (1.3%)	12,465,115	117,797 (0.9%)
36	4	–	6	–	1861	–
37	11	–	55	–	20,732	–
38	3	–	7	–	1774	–
39	2	–	2	–	108	–
40	1	–	1	–	2	–
41	1	–	1	–	97	–
42	1	–	1	–	10	–
43 *	11,169	82 (0.7%)	37,960	643 (1.7%)	9,309,934	89,794 (1.0%)
44	6	–	33	–	4276	–
45 *	23,331	183 (0.8%)	67,765	1306 (1.9%)	18,629,005	142,168 (0.8%)
46	3	–	7	–	5030	–
47	4	1 (25.0%)	6	1 (16.6%)	21,101	134 (0.6%)
48	3	–	12	–	8597	–
49	25	–	233	–	235,882	–
50	4	–	4	0 0.0%)	146	–
51 *	3534	2 (0.1%)	18,653	4 (0.02%)	5,750,091	221 (0.003%)
Total	74,082	364 (0.5%)	260,422	3142 (1.2%)	59,685,328	391,575 (0.6%)

**Table 3:** Collected device information for the Samsung Galaxy A10 Android phone, as well as, the count of corresponding phones in the NoiseCapture database.

‘device_model’	‘device_product’	‘device_manufacturer’	Count
SM-A102N	a10ekx	samsung	1
SM-A102U	a10esq	samsung	880
SM-A102U1	a10eue	samsung	1
SM-A102W	a10ecs	samsung	4
SM-A105F	a10dd	samsung	53
SM-A105FN	a10eea	samsung	176
SM-A105G	a10dx	samsung	31
SM-A105M	a10ub	samsung	97
SM-A107F	a10sxx	samsung	508
SM-A107M	a10sub	samsung	23

**Table 4:** Top 15 of smartphone manufacturers (‘device_manufacturer’, case insensitive) in the NoiseCapture database. The number of corresponding distinct device models (‘device_model’), the number of tracks, as well as the cumulative number of tracks are also given. Note that this table do not regroup data from the same manufacturer but with a different writing (upper/lower case, as for ‘Samsung’ and ‘samsung’).

Rank	Device_MANUFACTURER	Nb of Models	Nb of Tracks	%	Cumul. Nb of Tracks	%
1	samsung	1032	101,420	38.9%	101,420	38.9%
2	LGE	383	36,288	13.9%	137,708	52.9%
3	HUAWEI	454	31,937	12.2%	169,645	65.1%
4	motorola	126	17,822	6.8%	187,467	71.9%
5	ZTE	171	12,840	4.9%	200,307	76.9%
6	Xiaomi	106	6334	2.4%	206,641	79.3%
7	TCL	191	5180	2.0%	211,821	81.3%
8	Sony	167	5116	2.0%	216,937	83.3%
9	OPPO	91	3742	1.4%	220,679	84.7%
10	WIKO	73	3223	1.2%	223,902	86.0%
11	asus	115	2870	1.1%	226,772	87.1%
12	HTC	130	2406	0.9%	229,178	88.0%
13	HMD Global	41	2208	0.8%	231,386	88.8%
14	LENOVO	108	1881	0.7%	235,065	89.6%
15	OnePlus	32	1798	0.7%	235,065	90.3%

**Table 5:** Top 5 of smartphone model (‘device_model’) in the NoiseCapture database.

Rank	Device_MODEL	‘Device_MANUFACTURER’	Nb of Tracks	%	Cumulative Nb of Tracks	%
1	ANE-LX3	samsung	4729	1.8%	4729	1.8%
2	SM-G930F	samsung	3722	1.4%	8451	3.2%
3	LM-X210(G)	LGE	3479	1.3%	11,930	4.6%
4	SM-A520F	samsung	3205	1.2%	15,135	5.8%
5	Z982	ZTE	2890	1.1%	18,025	6.9%
6	SM-G935F	samsung	2854	1.1%	20,879	8.0%
7	Moto E (4)	motorola	2748	1.0%	23,627	9.1%
8	SM-N950U	samsung	2384	0.9%	26,011	9.9%
9	moto e5 play	motorola	2361	0.9%	28,372	10.9%
10	SM-G950F	samsung	2301	0.9%	30,673	11.8%
11	SM-J327T1	samsung	2297	0.9%	32,970	12.6%
12	LGMP260	LGE	2213	0.8%	35,183	13.5%
13	SM-S327VL	samsung	2200	0.8%	37,383	14.3%
14	VTR-L09	samsung	2095	0.8%	39,478	15.1%
15	SM-J727T1	HUAWEI	2048	0.8%	41,526	15.9%

**Table 6:** Top 6 smartphone manufacturers (‘device_manufacturer’, case-insensitive) for USA and France between August 2017 and August 2020. Top 6 manufacturers data are from Statcounter Global Stats website (licensed under a Creative Commons Attribution-Share Alike 3.0 Unported License) [40].

Country	Device_MANUFACTURER	Number of Tracks	%	Top 6 Manufacturer in Country
United States	samsung	32,341	36.6%	IPhone
	LGE	23,668	26.8%	Samsung
	motorola	9257	10.5%	LGE
	ZTE	8154	9.2%	Motorola
	TCL	2117	2.4%	Google
	Alcatel	1289	1.4%	ZTE
France	samsung	12,899	46.2%	Samsung
	HUAWEI	4864	17.4%	IPhone
	WIKO	1731	6.2%	HUAWEI
	Xiaomi	1294	4.6%	Sony
	Sony	1254	4.5%	Xiaomi
	motorola	1093	3.9%	WIKO

**Table 7:** Distribution of the number of contributors in function of the number of track measurements. The number of corresponding points is also given.

Number of Tracks	Number of Contributors	%	Number of Points	%
1	36,405	52.0%	8,709,872	14.6%
2–10	30,043	43.0%	24,106,578	40.1%
11–50	3063	4.4%	14,779,033	24.7%
51–100	236	0.3%	3,915,310	6.5%
101–1000	143	0.2%	8,016,517	13.4%
>1000	8	0.1%	158,018	0.7%
Total	69,898		59,685,328	

**Table 8:** Distribution of the time length (in second) in function of the number of track measurements for tracks collected by user who had 1 contribution to the database.

Time Length	Number of Tracks	%
1–20	14,034	38.5%
21–60	9452	26.0%
61–300	9242	25.4%
301–600	1537	4.2%
601–900	534	1.5%
901–1200	315	0.8%
1201–1800	368	1.0%
1801–2400	213	0.6%
2401–3000	111	0.3%
3001–3600	103	0.3%
>3600	496	1.4%
Total	36,405	

**Table 9:** Duration between two successive measurements for users who have more than one contribution.

Duration between 2 Successive Measurements (Day)	Number of Tracks	%
0	158,631	83.3%
1	7140	3.7%
2–7	11,831	6.2%
8–14	3911	2.0%
14–21	1923	1.0%
21–30	1450	0.8%
31–60	2256	1.2%
61–90	1063	0.6%
91–180	1269	0.7%
181–365	768	0.4%
>365	282	0.1%
Total	188,794	100%

**Table 10:** Distribution of the collected data per country and ranking (first ranks are displayed in bold). Population per country (percentage of world population) data are from in [43].

Country	Population (Rank)	Contributors (Rank)	Tracks (Rank)	Points (Rank)	Points/Track
China	**17.9% (1)**	58 (54)	354 (43)	158,797 (26)	448.6
India	17.5% (2)	894 (4)	2241 (16)	243,778 (22)	108.9
United States	4.2% (3)	**29,108 (1)**	**88,341 (1)**	**22,676,833 (1)**	256.7
Indonesia	3.4% (4)	91 (45)	199 (55)	20,244 (65)	101.7
Pakistan	2.8% (5)	124 (32)	321 (45)	27,035 (57)	84.2
Brazil	2.7% (6)	448 (14)	1503 (20)	244,482 (21)	162.6
Nigeria	2.7% (7)	33 (67)	66 (78)	9319 (75)	141.2
Bangladesh	2.1% (8)	160 (26)	572 (28)	332,316 (19)	581
Russia	1.86% (9)	174 (24)	850 (24)	94,277 (33)	110.2
Germany	1.1% (19)	790 (7)	3093 (7)	1,216,164 (5)	393.2
France	0.9% (20)	5516 (2)	27,911 (2)	4,972,054 (2)	178.1
United Kingdom	0.8% (21)	1164 (3)	4693 (4)	2,067,182 (3)	440.5
Canada	0.5% (37)	792 (6)	2512 (15)	1,551,808 (4)	**617.7**
Peru	0.4% (42)	77 (48)	11,231 (3)	138,716 (27)	12.3
Netherlands	0.2% (67)	435 (15)	3409 (5)	413,897 (16)	121.4

**Table 11:** Distribution of accuracy for the point with geolocalization.

‘accuracy’	Number of Points	%
0	0	0
$\left[0,1\right]$	420,150	0.9
$\left[1,2\right]$	1,400,466	2.8
$\left[2,3\right]$	3,574,004	7.3
$\left[3,4\right]$	7,406,060	15.1
$\left[4,5\right]$	5,268,843	10.8
$\left[5,10\right]$	11,142,305	22.8
$\left[10,15\right]$	5,973,000	12.2
$\left[15,25\right]$	7,128,773	14.6
$\left[25,35\right]$	1,725,169	3.5
$\left[35,50\right]$	1,192,908	2.4
$\left[50,100\right]$	1,426,475	2.9
>100	2,243,566	4.6
**Total**	48,901,719	

**Table 12:** Distribution of ‘speed’ for points with geolocalization.

‘speed’	Number of Points	%
0	31,986,102	65.4%
$\left[0,1.4\right]$	837,662	18.2%
$\left[1.4,4.2\right]$	2,058,585	4.2%
>4.2	5,919,370	12.2%
Total	48,901,719	

**Table 13:** Time shift (in hour) between record_utc and time_location, for the tracks are are 100% geolocalized.

Time Shift (h)	Number of Tracks	%
<−24	797	0.4
−24	151	0.07
−23	35	0.02%
−22	4	0.002%
−20	3	0.002%
−19	8	0.004%
−18	8	0.004%
−15	1	0.0005%
−14	1	0.0005%
−13	3	0.002%
−12	25	0.012%
−11	17	0.008%
−10	8	0.004%
−9	6	0.003%
−8	3	0.002%
−7	10	0.005%
−6	7	0.004%
−5	6	0.003%
−4	10	0.005%
−3	67	0.034%
−2	47	0.023%
−1	258	0.13%
0	194,690	98.54%
1	479	0.242
2	54	0.027%
3	28	0.013%
4	15	0.008
5	10	0.005%
6	6	0.003%
7	6	0.003%
8	10	0.005%
9	3	0.002%
10	10	0.005%
11	4	0.002%
12	1	0.0005%
13	3	0.002%
14	2	0.001%
15	8	0.004%
16	11	0.006%
18	8	0.004%
19	4	0.002%
20	5	0.002%
21	2	0.001%
22	3	0.002%
23	18	0.009%
24	11	0.006%
>24	702	0.355%
**Total**	197,568	

**Table 14:** Total number of collected data and distribution per time length and part of the measurement tracks and points that have been collected in motion (only data with geolocalization are considered, with a speed greater than 0.5 m/s).

‘time_length’	Tracks		Points	
	Total	Moving	Total	Moving
$\left[1,3\right]$ min	21,963	4477 (20.4%)	679,675	437,640 (64.4%)
$\left[3,5\right]$ min	7190	1575 (21.9%)	516,389	343,035 (66.4%)
$\left[5,10\right]$ min	7204	2029 (28.16%)	1,080,596	769,976 (71.2%)
$\left[10,20\right]$ min	5231	1678 (32.1%)	1,661,753	1,189,641 (71.6%)
$\left[20,30\right]$ min	2083	752 (36.1%)	1,237,893	925,499 (74.7%%)
$\left[30,60\right]$ min	2362	741 (31.4)	2,094,625	1,446,091 (69.0%)
$\left[1,5\right]$ h	2026	466 (22.9%)	3,694,683	1,912,894 (51.7%)
>5 h	142	19 (13.4%)	752,485	189,075 (25.1%)

**Table 15:** Distribution of tracks in function of the calibration method, before and after release 49 (’n.a.’ for ‘not available’).

‘calibration_method’	Since Release	Nb of Tracks before R49 (%)	Nb of Tracks Since R49 (%)
CalibratedSmartPhone	33	n.a.	277 (1.4%)
Calibrator	28	n.a.	139 (0.7%)
ManualSetting	28	n.a.	838 (4.4%)
None	28	241,532	17,395 (92.1%)
Reference	28	n.a.	167 (0.9%)
Traffic	49	n.a.	74 (0.5%)
Total		241,532	18,890

**Table 16:** Cross table of the number of collected tracks in function of the calibration method and the calibration gain (data from release 49).

Calibration Method/Gain	<−10 dB	[−10, −5] dB	[−5, 0] dB	0 dB	[0, 5] dB	[5, 10] dB	>10 dB	Total
CalibratedSmartPhone	-	61 (22.0%)	136 (49.1%)	-	75 (27.1%)	3 (1.1%)	2 (0.7%)	277
Calibrator	1 (0.7%)	2 (1.4%)	3 (2.2%)	-	7 (5.0%)	-	126 (90.7%)	139
ManualSetting	56 (6.7%)	13 (1.6%)	150 (17.9%)	126 (15.%)	156 (18.6%)	54 (6.4%)	283 (33.8%)	838
None	559 (2.4%)	911 (5.3%)	865 (5.1%)	13,857 (79.9%)	485 (2.9%)	227 (1.5%)	491 (2.9%)	17,395
Reference	9 (5.4%)	21 (12.6%)	38 (22.7%)	23 (13.8%)	57 (34.1%)	7 (4.2%)	12 (7.2%)	167
Traffic	20 (27.0%)	14 (18.9%)	28 (37.8%)	-	6 (8.1%)	5 (6.8%)	1 (1.4%)	74
Total	645	1022	1220	14,006	786	296	915	18,890

**Table 17:** List of NoiseCapture Parties. More information are located in the ‘noisecapture_party’ table of the database. The number of contributors, as well as the total of collected tracks and points are given. The organization in charge of the NoiseCapture Party is also mentioned (Noise-Planet is the organization in charge of the development of NoiseCapture application). While the CICAM NoiseCapture Party has been planned, it was canceled due to the pandemic situation (i.e., there are no corresponding tracks).

‘pk_party’	‘tag’	Organization	‘filter_area’	‘filter_time’	Contributors	Tracks	Points
1	SNDIGITALWEEK	Noise-Planet	TRUE	FALSE	7	133	11,523
2	ANQES	Noise-Planet	TRUE	TRUE	4	29	4479
3	FDS2017	Noise-Planet	TRUE	TRUE	2	6	1239
5	IMS2018	Noise-Planet	TRUE	FALSE	13	67	18,793
6	UDC	Universidade da Coruña	TRUE	TRUE	8	56	6879
9	TEST44	Noise-Planet	TRUE	TRUE	1	3	91
10	UNISA	University of Salerno, Italy	TRUE	TRUE	13	149	15,912
11	PNRGM	Noise-Planet	TRUE	TRUE	2	13	6089
12	AMSOUNDS	Waag Technology & Society	TRUE	TRUE	2	18	693
13	PNRGM	Parc Naturel du Morbihan	TRUE	TRUE	14	100	21,470
14	FDSSTRAS	Noise-Planet	TRUE	TRUE	5	31	2967
15	AGGLOBASTIA	Noise-Planet	FALSE	TRUE	19	507	59,838
17	FDSNTS	Noise-Planet	TRUE	TRUE	7	66	5916
18	H2020	Noise-Planet	TRUE	TRUE	11	89	22,060
19	UDC	Universidade da Coruña, Spain	FALSE	TRUE	20	138	5866
20	MSA	Noise-Planet	TRUE	TRUE	9	9	1885
21	GEO2019	Noise-Planet	TRUE	TRUE	43	420	63,521
22	IMS2019	Noise-Planet	TRUE	TRUE	23	192	17,309
23	FPSLYO	Noise-Planet	TRUE	TRUE	11	34	10,285
24	SSSOROLL2019	Generalitat de Catalunya	FALSE	TRUE	68	372	36,272
26	UNISA	University of Salerno, Italy	TRUE	TRUE	20	332	23,220
27	FDSSTRAS	Noise-Planet	TRUE	TRUE	3	7	1771
28	H2020	Noise-Planet	TRUE	TRUE	9	39	32,948
29	UDC	Universidade da Coruña, Spain	FALSE	TRUE	9	73	2099
30	MSA	Noise-Planet	TRUE	TRUE	10	10	3665
31	CICAM	EPN, Quito, Ecuador	FALSE	TRUE	-	-	-
32	UDC_COVID	Universidade da Coruña, Spain	TRUE	TRUE	33	249	14,785

**Table 18:** Cross table between pleasantness and tags.

Tag/Pleasantness	Used	Not Used
**Used**	45,549 (17.5%)	78,814 (30.2%)
**Not used**	9457 (3.6%)	126,602 (48.7%)

**Table 19:** Tags description: ‘pk_tag’ and ‘tag_name’ are the primary key and the name of the tags. The ‘Description’ correspond to the name of the tag in the corresponding NoiseCapture screen.

**Category**	**Measurement Conditions**			
‘pk_tag’	1	6	13	23
‘tag_name’	test	indoor	rain	wind
Description	Test	Indoor	Rain	Wind
	**Human Activity Sources**			
‘pk_tag’	18	30	20	28
‘tag_name’	chatting	children	footsteps	music
Description	Voice	Children	Footsteps	Music
**Category**	**Transportation Sources**			
‘pk_tag’	27	32	26	35
‘tag_name’	road	rail	air_traffic	marine_traffic
Description	Road	Rail	Air T.	Marine
	**Natural Sources**			
‘pk_tag’	34	33	29	
‘tag_name’	water	animals	vegetation	
Description	Water	Animals	Vegetation	
	**Mechanical Activity Sources**			
‘pk_tag’	24	36	31	
‘tag_name’	works	alarms	industrial	
Description	Works	Alarms	Industrial	

**Table 20:** Possible enhancements of the NoiseCapture application and database.

Data	Uncertainties/Bias	Possible Sources	Possible Solutions
User profile	Profile information is empty	Cannot evaluate the expertise of the contributor	In the app: update the field during an app update if the field is empty.
Geolocalization	No geolocalization of a track	The geolocalization is turned off	In the app: add a message for turning on the geolocalization
		Indoor measurements	In the app: wait for future methodologies (Indoor positioning System) and high sensitivity GPS for indoor localization
	No geolocalization of a point in a track	Local loss of geolocalization	Use GIS methodologies to re-locate the point within the track
	Inhomogeneous worldwide coverage	No access to Google Play	Use alternative app stores
Accuracy	Value equal to ‘0.0’	No geolocalization	In the app: add a message for turning on the geolocalization
	Extreme (not realistic) values	Unknown	No known solutions
	Large (but realistic) values		In the app: ask contributors to wait for a better localization before starting the measurement
Speed	Value equal to ‘0.0’	No geolocalization	In the app: add a message for turning on the geolocalization
	Negative values	Unknown	No known solutions
		No evaluation of the accuracy of the speed value	In the app: use the Android function getSpeedAccuracyMetersPerSecond() to store this missing information.
Timestamp	Wrong date	The geolocalization is turned off	In the app: add a message for turning on the geolocalization
		Wrong phone setting	In the app: check that the date is correct and add a message if not
Calibration	The calibration method is not known	The information about the selected calibration method is collected since the version 49 only	No solution
	Extreme (not realistic) values	No calibration method used	In the app: send a notification to calibrate the smartphone
			In the app: check the calibration value and send a notication if the value seems incorrect
			In the app/remote server: create a smartphone model calibration database
Pleasantness	Possible bias at level 50%	The default value is fixed at a pleasantness of 50%	In the app: change the selection mode for the pleasantness without default value
Noise levels	Extreme (not realistic) values	Calibration is not correct	Improve the calibration of the smartphone

## Data Availability

The data presented in this study are openly available from Université Gustave Eiffel Dataverse Repository at https://doi.org/10.25578/J5DG3W.

## Abbreviations

The following abbreviations are used in this manuscript:

API	Application Programming Interface
CNRS	Centre national de la recherche scientifique (French National Centre for
	Scientific Research)
ERD	Entity relation diagrams
GPS	Global positioning system
ICT	Information and communication Technologies
Ifsttar	Institut Français des sciences et technologies des transports, de l’aménagement et
	des réseaux
OSM	OpenStreetMap
RNE	Repartition of the noise exposure
SDI	Spatial data infrastructure
SPL	Sound Power Level
